# Large-scale gene expression study in the ophiuroid *Amphiura filiformis* provides insights into evolution of gene regulatory networks

**DOI:** 10.1186/s13227-015-0039-x

**Published:** 2016-01-11

**Authors:** David Viktor Dylus, Anna Czarkwiani, Josefine Stångberg, Olga Ortega-Martinez, Sam Dupont, Paola Oliveri

**Affiliations:** Research Department of Genetics, Evolution and Environment, University College London, Room 426, Darwin Building, Gower Street, London, WC1E 6BT UK; CoMPLEX/SysBio, UCL, Gower Street, London, WC1E 6BT UK; Department of Biological and Environmental Sciences, Sven Lovén Centre for Marine Sciences, University of Gothenburg, Kristineberg 566, 451 78 Fiskebäckskil, Sweden; Department of Ecology and Evolution, University of Lausanne, 1015 Lausanne, Switzerland; Research Department of Animal Ecology, Evolutionary Biology Centre, Uppsala University, Norbyvägen 18D, 752 36 Uppsala, Sweden

**Keywords:** Echinoderms, Brittle star, *Amphiura filiformis*, Skeleton, Gene regulatory network, Evolution

## Abstract

**Background:**

The evolutionary mechanisms involved in shaping complex gene regulatory networks (GRN) that encode for morphologically similar structures in distantly related animals remain elusive. In this context, echinoderm larval skeletons found in brittle stars and sea urchins provide an ideal system. Here, we characterize for the first time the development of the larval skeleton in the ophiuroid *Amphiura filiformis* and compare it systematically with its counterpart in sea urchin.

**Results:**

We show that ophiuroids and euechinoids, that split at least 480 Million years ago (Mya), have remarkable similarities in tempo and mode of skeletal development. Despite morphological and ontological similarities, our high-resolution study of the dynamics of genetic regulatory states in *A. filiformis* highlights numerous differences in the architecture of their underlying GRNs. Importantly, the *A.filiformispplx*, the closest gene to the sea urchin double negative gate (DNG) repressor *pmar1*, fails to drive the skeletogenic program in sea urchin, showing important evolutionary differences in protein function. *hesC*, the second repressor of the DNG, is co-expressed with most of the genes that are repressed in sea urchin, indicating the absence of direct repression of *tbr*, *ets1/2*, and *delta* in *A. filiformis*. Furthermore, the absence of expression in later stages of brittle star skeleton development of key regulatory genes, such as *foxb* and *dri*, shows significantly different regulatory states.

**Conclusion:**

Our data fill up an important gap in the picture of larval mesoderm in echinoderms and allows us to explore the evolutionary implications relative to the recently established phylogeny of echinoderm classes. In light of recent studies on other echinoderms, our data highlight a high evolutionary plasticity of the same nodes throughout evolution of echinoderm skeletogenesis. Finally, gene duplication, protein function diversification, and *cis*-regulatory element evolution all contributed to shape the regulatory program for larval skeletogenesis in different branches of echinoderms.

**Electronic supplementary material:**

The online version of this article (doi:10.1186/s13227-015-0039-x) contains supplementary material, which is available to authorized users.

## Background

The genome is a biological instruction manual and its regulatory program is executed by large gene regulatory networks (GRN) that causally explain the connection between genotype and phenotype. Body plans and complex morphological structures arise during embryogenesis and rely on the expression of a distinct array of regulatory genes (i.e., genes encoding transcription factors (TF) and proteins of signaling pathways). Understanding how developmental GRNs change during evolution will directly contribute to addressing the fundamental question of how specific DNA sequence variations lead to different morphologies.

In development, GRNs describe the progression of regulatory states (generated by a unique combination of transcription factors) in embryonic space and time, necessary to specify different cell types present in a multicellular organism [1, 2]. Although many studies in different organisms have analyzed complex developmental GRNs [1, 3–5], little is known about the mechanisms of rewiring during evolution, and most studies, with few exceptions [6–8], remain at the level of single nodes. Comparative analyses in the past two decades suggest that phenotypic differences between organisms are achieved mostly through variation in the expression of developmental regulatory genes [9]. Therefore, changes in the *cis*-regulatory apparatuses that control gene expression act as the main mechanism of GRN evolution [1, 8–13]. Alterations in *cis*-regulatory elements represent only one aspect of GRN evolution. Important evolutionary changes of transcription factors have been reported also at the level of functional domains and binding specificity (for review [14, 15]).

Echinoderms and their calcitic endoskeleton provide an ideal system to study mechanisms of GRN evolution due to the extensive knowledge of GRNs for several sea urchin species [2, 16–19]. In the euechinoid sub-class of sea urchins, the cells producing the larval skeleton are precociously segregated at early cleavage stage in the form of four small cells at the vegetal pole (large micromeres). At this stage, the skeletogenic program is strictly activated by a cascade of localized repressors (*pmar1* and *hesC*), the so-called double negative gate (DNG), which allows the expression of a cohort of regulatory genes in these cells. The activated regulatory genes are precisely wired to ensure progression of the genetic program up to the expression of the differentiation genes, which produce the bio-mineralized skeleton of the pluteus larva [2, 16, 17]. Recent studies on the Cidaroidea sub-class of sea urchins, which also develop a larval skeleton showed, however, major differences in the initiation of the skeletogenic GRN and can be summarized as follows: (1) the absence of a clear precocious segregation of the skeletogenic lineage at cleavage stage; (2) the absence of a clear *pmar1* or similar functioning gene; and (3) the variability of expression and function of *hesC* in different cidaroid species [18, 19]. Furthermore, a clear delay in the ingression of the skeletogenic cell lineage at late gastrula stage is characteristic of the cidaroid skeleton development [20]. Little is known about the rest of the network components and linkages in cidaroid sea urchins.

Of the five extant classes of echinoderms, only echinoids (sea urchins) and ophiuroids (brittle stars) develop an elaborated larval skeleton. A gene expression study of sea urchin and sea star adult skeletogenesis hypothesized that sea urchin larval skeleton originated by co-option of an existing regulatory module into a new developmental context [21]. An open question remains: how did the brittle star larval skeleton originate?

To address the directionality of evolutionary changes, the phylogenetic relationship of echinoderm classes is of paramount importance. While morphology and molecular analysis agree on a monophyletic group of asteroids, ophiuroids, echinoids, and holothurians (the Eleutherozoa), molecular evolution studies sustain two alternative positions of the ophiuroids within the Eleutherozoa: (1) sister group to asteroids [22–25] or (2) sister group to echinoids plus holothurians [26]. Although this question is still debated, four recent independent studies showed clear support for ophiuroids as sister group to asteroids [22–25].

Independent of their phylogenetic relationships, it is clear that ophiuroid and echinoid lineages split around 480 million years ago (Mya) [23, 26] and the comparison of development of the larval skeleton will highlight changes and conservation of the GRN responsible for the formation of this complex structure. Additionally, these studies may help elucidate the common or independent origin of the pluteus larvae and their extended skeleton in echinoids and ophiuroids, a question still subject to debate.

The brittle stars (Ophiuroidea), along with sea lilies (Crinoidea), are the least studied class of Eleutherozoa for molecular aspects of their development. This is largely caused by experimental difficulties, mostly due to the inability to control oocyte maturation [27], a short spawning season, a small number of gametes per adult, and an often deep-water habitat. Furthermore, so far no functional experiments have been successfully performed, making this system extremely challenging. The few studies that have been performed on brittle star development are scattered among various species. Although superficially similar to the sea urchin pluteus larva, the embryo of ophiuroids differ in the lack of micromeres [28] and the pluteus shows greater sensitivity to ocean acidification [29]. On the other hand, a transcriptome analysis identified several ophiuroid orthologs of sea urchin skeletogenic genes [30], and expression of *ets1/2,vegfR*, and *vegf* regulatory genes [31, 32] showed remarkable resemblance to sea urchin, suggesting a similar molecular make-up of the skeleton. While brittle stars are experimentally challenging, their key phylogenetic position is ideal to answer important questions about the evolution of the skeleton in echinoderms and the corresponding regulatory network encoding it.

Here, we are using a multi-gene approach to compare development and gene expression between the brittle star *Amphiura filiformis* (Afi) and the sea urchins, using mainly, but not exclusively *Strongyloncentrotus purpuratus* (Spu) as an entry to understand GRN evolution [2]. Detailed characterization of *A. filiformis* development identified a group of cells marked by a skeletogenic molecular signature emerging as early as blastula stage. Our data show major changes in the initiation of a specification network subcircuit (i.e., the *pmar1/hesc* double negative gate) as well as the complete absence of expression of late regulatory genes (*foxb* and *dri*) in *A. filiformis*, and the heterochronic change in the expression of several other genes. In summary, these data indicate major differences in GRN architecture for larval skeletogenesis in these two classes, which suggest that gene duplication, protein function diversification, and *cis*-regulatory elements evolution all contributed to shape the regulatory program for larval skeletogenesis in different classes of echinoderms.

## Results

### Features of *Amphiura filiformis* embryonic development and skeletogenesis

**Fig. 1:**
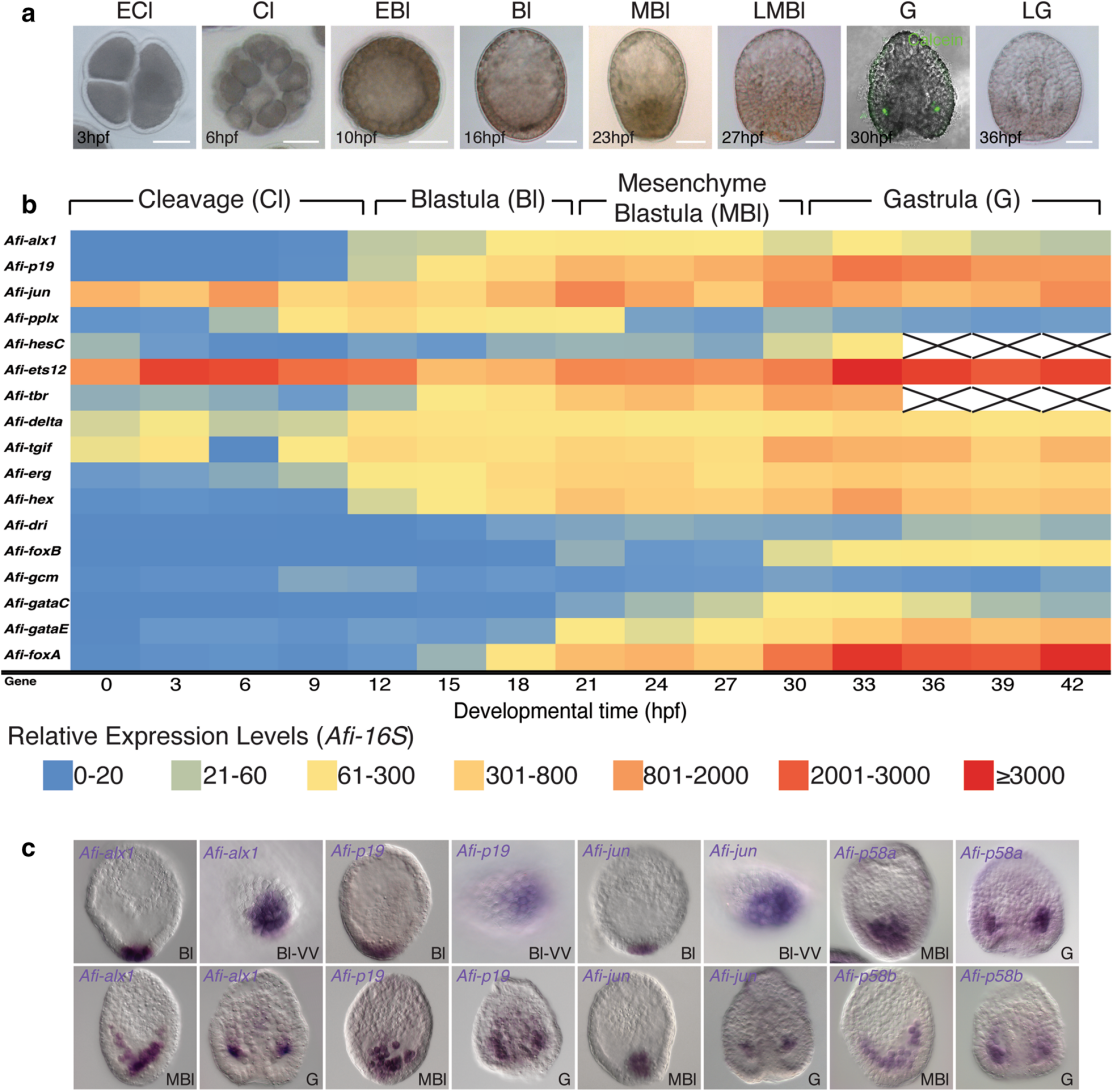
*Amphiura filiformis* development and identification of skeletogenic cells. **a** Live imaging of embryos. Early cleavage stage shows tetrahedral arrangement of cells at 3 hpf, similar to *Ophiopholis aculeata* [28]. Mid-cleavage stage shows equally sized cells at 6 hpf. Early blastula stage shows spherical embryo with blastocoel at 10 hpf. Hatched blastula embryos have distinct blastocoel at 16 hpf and animal–vegetal orientation is visible through thickening at vegetal side of the embryo. At 23 hpf, mesenchyme blastula stage shows first ingressing cells from the vegetal side of the embryo that fill up the blastocoelar space by 27 hpf. At 30 hpf, calcein (*green*) stained gastrula embryo shows two newly formed spicules that extent to a tri-radiate structure as visible on a bright field by 36 hpf. **b** High-resolution time-courses for genes analyzed in this study shown as heatplot were obtained by QPCR. Expression values are relative to *Afi*-*16S* (see Additional file 1 for explanation of calculation and QPCR controls; exact numbers are shown in Table S1). **c** WMISH of skeletogenic marker genes identifies the vegetal plate and the primary ingressing cells as the SM cell lineage in *A. filiformis*. The two regulatory genes *Afi*-*alx, Afi*-*jun* and the skeleton matrix gene, *Afi*-*p19,* are expressed in the vegetal plate of the blastula embryos, then in the first ingressing mesenchymal cells and at later stage in a location congruent to where spicules are formed. Other two orthologs of sea urchin skeletogenic matrix genes, *Afi*-*p58a* and *Afi*-*p58b,* are also detected in the first ingressing cells of the mesenchyme blastula stage and at later stage in the same location where the spicules are formed. *ECl* early cleavage, *Cl* cleavage, *EBl* early blastula, *Bl-VV* blastula vegetal view, *Bl* blastula, *MBI* mesenchyme blastula, *LMBl* late mesenchyme blastula, *G* gastrula, *LG* late gastrula. *Scale bars* are 50 μm

In this study, we compare development and gene expression between the brittle star *Amphiura filiformis* and indirectly developing echinoids with elaborated skeleton [2, 18, 19]. To understand larval skeletogenesis in ophiuroids, we first analyzed the tempo and the mode of *A. filiformis* development, providing the most complete picture of ophiuroid development to date (Fig. 1). *A. filiformis* differs from euechinoid sea urchin development in the lack of micromeres at the vegetal pole (Fig. 1a, 6hpf), and in the early appearance of morphological evidence for animal–vegetal and oral–aboral axes (Fig. 1a, 16hpf and 23hpf). The fertilized egg undergoes 8 rounds of cleavages in the first 10 h of development producing a blastula of approximately 250 cells with a clearly visible blastocoel and still encased in the fertilization membrane (Fig. 1a, 10hpf ). Immediately after hatching (Fig. 1a, 16 hpf), the embryos elongate along the animal–vegetal axis and the cells in the vegetal half are distinctly thicker than the ones in the animal half. At the beginning of gastrulation (Fig. 1a, 27 hpf), *A. filiformis* embryos flatten along the oral–aboral axis. Furthermore, morphogenetic movements occur at a faster pace, although following a similar sequence of events (Fig. 1). Despite these differences, in both organisms skeletogenesis is preceded by the ingression of mesenchymal cells prior to gastrulation (Fig. 1a, 23 hpf), and the two bilaterally arranged spicules are formed just underneath the ectoderm within two clusters of mesenchymal cells located at the boundary with the invaginating endoderm as identified by calcein staining (Fig. 1a, 30 hpf) (for comparison with sea urchin see [33]).

Skeleton-specific genes, therefore, should be expressed in the cells tightly associated with skeletal elements in the two lateral patches of the *A. filiformis* gastrula where the spicules appear. We selected genes whose zygotic expression is present exclusively in skeletogenic cells throughout development in sea urchin and cloned their orthologs in *Amphiura*. These genes are encoding for transcription factors, such as *alx1*, *jun* [17, 34], and for differentiation proteins identified by proteomic studies in the bio-mineralized matrix of both larval and adult skeleton, such as *p19*, *p58a* and *p58b* [35–37]. Using whole mount in situ hybridization (WMISH), we observe their expression in the cells where *A. filiformis* skeleton primordia first appear (Fig. 1c). The expression of these genes remains associated only with the growing skeleton also later in development, as shown by the staining in the two mesenchymal clusters of cells at the base of the archenteron throughout gastrulation, where the skeleton becomes evident, and in a chain of mesenchymal cells distributed in a pattern that mirrors the elaborated skeletal structure of the pluteus (Additional file 1: Figure S1). Similar to sea urchin, these genes are exclusively expressed in cells surrounding the position of skeleton formation. Moreover, their co-expression over developmental time allows us to use their combination here as a marker for skeletogenesis. To establish the onset of expression of these skeletogenic lineage specific genes, we analyzed high-resolution time-courses using QPCR (Fig. 1b). *Afi*-*alx1* and *Afi*-*p19* start to be expressed at early blastula (12 hpf), while the maternally abundant *Afi*-*jun* decreases in the first 9 h of development until its zygotic expression is activated at 12 hpf (Fig. 1b). WMISH of *Afi*-*alx1* at early blastula shows expression in 8 (±1) cells (n = 3) grouped together on one side of the embryo (Fig. 1c, Additional file 1: Figure S2) and is later expressed in 18 (±3) cells (n = 17) in the vegetal plate of the late blastula (Fig. 1c). Importantly, none of these three genes show any localized expression at earlier stages of development (Additional file 1: Figure S2), consistent with the QPCR data. At early blastula stage, next to similar cell counts, double fluorescent in situ hybridization (FISH) shows complete co-expression of *Afi*-*alx1* and *Afi*-*p19* (Additional file 1: Figure S2C). At mesenchyme blastula stage staining for these genes, as well as *Afi*-*p58a* and Afi-*p58b*, is detected only in the mesenchymal cells ingressing first, and later at gastrula stage in cells surrounding the position of calcium carbonate deposition (compare Fig. 1a, c). Taken together these high-resolution expression data suggest that, as in euechinoids, the primary mesenchyme cells might be the precursors of the skeletal cells and are here referred to as skeletogenic mesodermal cells (SM). These data also identify a specific regulatory program already present at blastula stage in a subset of the vegetal cells of the *A. filiformis* embryo. This is characterized by the presence of the transcription factors *Afi*-*alx1* and *Afi*-*jun* and their co-expression with the differentiation genes *Afi*-*p19*, *Afi*-*p58a* and *Afi*-*p58b* for which sea urchin orthologs are participating in the formation of the bio-mineral matrix of the skeleton. The exact function of these genes in *A. filiformis* development and their role in the formation of the skeleton need to be tested in knock-down experiments in future investigations.

### Functional differences in the *A. filiformis* ortholog of the sea urchin skeletogenic initiator gene

**Fig. 2:**
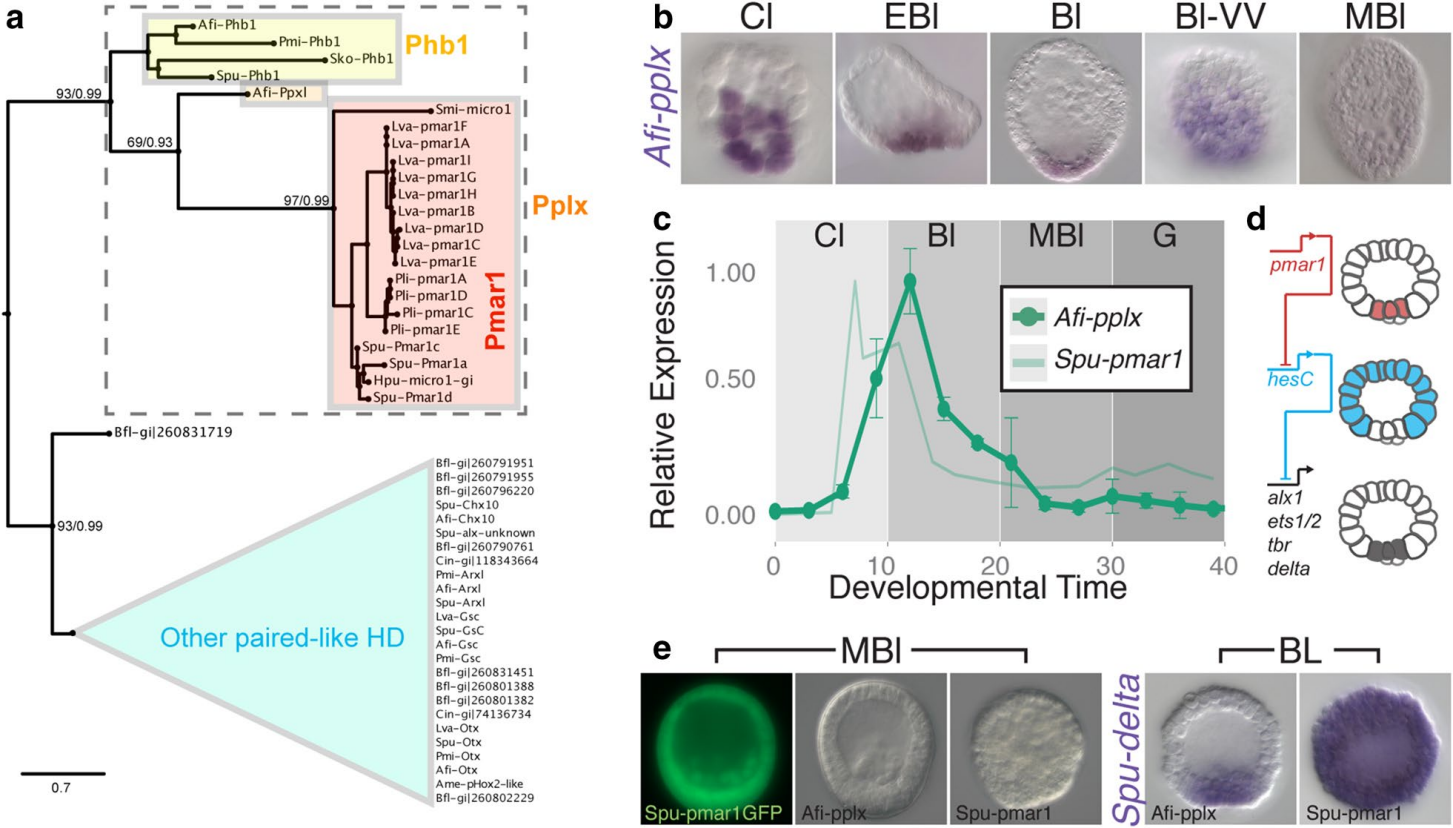
*Afi*-*pplx* is expressed similar to sea urchin *Spu*-*pmar1*, but does not function as repressor. **a** Phylogeny of Pplx and Pmar1 proteins suggesting orthology of these genes. Other paired-like homeodomains are use as outgroup. First value on branch is bootstrap support and second value is posterior probability. Tree is the consensus of differently constructed trees, using different initial alignments as well as different methodologies (Additional file 1: Figure S3). **b** WMISH showing the expression of *Afi*-*pplx* during *A. filiformis* development. **c** Time-line comparison of *Spu*-*pmar1* and *Afi*-*pplx* transcript abundance adjusted for the stages of development (see Additional file 1: Figure S10) and normalized to their individual maximum of expression shows high correlation of expression dynamic (cross-correlation: 0.801). For brittle star, error bars represent standard deviation of two biological replicas. Sea urchin data were obtained from [59]. **d** Schematic representation of the double negative gate in euechinoid, showing how the large micromeres are specified to be skeletogenic. **e** *S. purpuratus* embryos injected with synthetic mRNA for *Spu*-*pmar1*, *Afi*-*pplx*, and GFP control (see Additional file 1: Figure S4 for details). No phenotype is observable in *Afi*-*pplx*-mRNA embryos or GFP controls, while injection of *Spu*-*pmar1*-mRNA induces skeletogenic fate in all cells. WMISH of *Spu*-*delta* in embryos injected with *Afi*-*pplx*-*mRNA* or *Spu*-*pmar1*-*mRNA*, which show expansion of *Spu*-*delta* expression to the whole embryo in *Spu*-*pmar1*-*mRNA*-injected embryos. *Bfl* *Branchiostoma floridae,* *Lva* *Lytechinus variegatus*, *Pli* *Paracentrotus lividus*, *Hpu* *Hemicentrotus pulcherrimus*, *Spu* *Strongyloncentrotus purpuratus*, *Afi* *Amphiura filiformis*, *Pmi* *Patiria miniata*, *Sko* *Saccoglossus kowalevskii*, *VV* vegetal view, *Cl* cleavage, *EBl* early blastula, *Bl* blastula, *MBl* mesenchyme blastula

In euechinoid sea urchin, the skeletogenic program is initiated by the zygotic expression of the paired-like homeodomain transcriptional repressor *pmar1/micro1*, which starts to be expressed only in micromeres as early as 4th cleavage [38, 39]. Pmar1 dominantly represses the globally expressed *hesC* and separates the skeletogenic lineage from the rest of the embryo forming the first element of the DNG (Fig. 2c). A potential brittle star *pmar1* has been mentioned in the study of the *Ophiocoma wendtii* developmental transcriptome [30]. Using a reciprocal blast approach in an *A. filiformis* developmental transcriptome, which includes four developmental time-points from cleavage to gastrula stage, we identified a sequence with closest similarity to *Spu*-*pmar1c,* here referred to as *Afi*-*pplx*. We validated its true evolutionary relationship with the *pmar1* genes through maximum likelihood and bayesian phylogenetic trees in the context of several classes of paired-like homeodomain (HD) sequences identified in different echinoderms and non-vertebrate deuterostomes. Using an alignment of the HD alone and independent of methodology, Afi-Pplx always grouped with good support as sistergroup of euchinoid Pmar1/Micro1 transcription factors (posterior probability of 0.93 and bootstrap of 63; Fig. 2a, Additional file 1: Figure S3A, C). On the other hand, the inclusion of other conserved domains in our analysis of the Pmar1/Micro1 proteins (i.e., engrailed repressor domains, eh1), resulted either in a highly supported polychotomy of Afi-Pplx, Phb1, and Pmar1/Micro1 genes using Bayesian inference (posterior probability 0.99; Additional file 1: Figure S3B) or in a low supported independent grouping of Afi-Pplx with brittle star and sea star (*Patiria miniata*, Pmi) Phb1 genes, whereas Pmar1/Micro1 genes group with Spu-Phb1 (bootstrap <60; Additional file 1: Figure S3D). Interestingly, all trees supported a monophyletic grouping of Afi-Pplx with Phb1 and Pmar1/Micro1 genes with high confidence (posterior probability 0.99 and bootstrap >83; Fig. 2a, Additional file 1: Figure S3). For this reason, we decided to name the Afi gene *pmar1*-*phb1*-*like*-*homeobox* (*pplx*). Interestingly, we were unable to find any close *pmar1* hit in the available cidaroid (*Eucidaris tribuloides*), sea star (*Patiria miniata*) and hemichordate (*Saccoglossus kowalevskii*) genomes, although a *phb1*-related sequence was present. Our phylogeny clearly reveals (1) the *phb1* and the *pmar1* + *pplx1* genes form a strongly supported distinct class of paired-like homeodomain; (2) the long branch to the euechinoid *pmar1/micro1* genes and the absence of a clear cidaroid *pmar1* gene suggest a recent evolution of these genes only in euechinoids; and (3) in euechinoids the *pmar1* genes have been extensively duplicated.

Importantly, the temporal and spatial expression of *Afi*-*pplx* is highly similar to the sea urchin *Spu*-*pmar1* (Fig. 2b, c). *Afi*-*pplx* is transiently expressed only in the zygote, starting its expression at late cleavage in a group of 10 (±3) cells (n = 7). It has a maximum level of expression at early blastula, when it is expressed in 20 (±5) cells (n = 4), then in 35 (±6) cells (n = 7) at the vegetal plate, to drop down to undetectable levels by mesenchyme blastula (Fig. 2b, c). It is important to notice that the number of cells expressing *Afi*-*pplx* at blastula stage is twice as much as the number of SM cells marked by *Afi*-*alx1*. A detailed analysis of protein domains using sequence comparison revealed that Afi-Pplx lacks two eh1 motifs, which are necessary for the Spu-Pmar1 repressive function [40]. It has been shown that this short protein motif can easily be acquired and lost throughout evolution [41]. In Afi-Pplx, moreover, amino acid (aa) 50 of the homeodomain, known as the recognition aa, is an H instead of a Q (Additional file 1: Figure S4A). On this basis, we hypothesize that Afi-Pplx is not functionally similar to Spu-Pmar1 and unlikely acts as transcriptional repressor, despite a similar domain of expression. To test this, we injected sea urchin fertilized eggs with equimolar amount of a synthetic mRNA encoding for Afi-Pplx and Spu-Pmar1 as already described [40]. We also injected a GFP only as negative control for injection artifacts (Spu-5′pmar1-gfp; Fig. 2e, Additional file 1: Figure S4) and controlled for correct translation of the synthetic Afi-pplx mRNA using a fusion with GFP (Afi-pplx-gfp; Additional file 1: Figure S4A), which indeed is translated and localized in all nuclei of the sea urchin embryos (Additional file 1: Figure S4 B). Whereas ectopic expression of *Spu*-*pmar1* leads to the re-specification of every cell of the embryo to a skeletogenic fate [40], ectopic expression of *Afi*-*pplx* does not show any re-specification of cells towards the skeletogenic fate, as shown also at molecular level with the lack of expansion of *Spu*-*delta* expression (Fig. 2e). To better characterize at molecular level the effects of *Afi*-*pplx* injection on the sea urchin skeletogenic program and exclude any potential compensatory effects, we quantified the level of expression of ten sea urchin skeletogenic genes, using QPCR, in *Afi*-*pplx*- and *Spu*-*pmar1*-injected embryos and compared them with GFP-injected controls. The sea urchin genes analyzed include all the immediate downstream genes of the double negative gate (*Spu*-*delta*, *Spu*-*tbr*, *Spu*-*ets1/2*, and *Spu*-*alx*), as well as late specification genes (*Spu*-*foxb*), signaling receptor (*Spu*-*vegfr*), and skeleton matrix genes (*Spu*-*sm50*, *Spu*-*p19*, *Spu*-*p58a*, *Spu*-*p58b*). In agreement to what has been already published by [2], the *Spu*-*pmar1*-injected embryos show upregulation of all skeletogenic genes above threshold levels (ΔΔCt > 1.6); on the contrary, *Afi*-*pplx*-injected embryos show little or no effect on all genes analyzed (Additional file 1: Figure S4F). Our results indicate that *Afi*-*pplx* is not capable of repressing the *Spu*-*hesC* gene and, thus, operates differently from *Spu*-*pmar1*. Interestingly, ectopic expression of *Afi*-*pplx*-mRNA shows specific and reproducible phenotypic effects on the development of the *S. purpuratus* skeleton at a later stage (Additional file 1: Figure S4D, E). This could be the result of Afi-Pplx having an activator function, opposite to Pmar1 repression, or a consequence of different interactions of these two transcription factors with other regulatory partners and/or *cis*-regulatory sequences. In summary, these data suggest that although *Afi*-*pplx* is expressed in a very similar spatio-temporal pattern of *Spu*-*pmar*, it might provide a different regulatory function in the brittle star skeletogenic program.

### In *A. filiformis* HesC is unlikely to be a repressor of *delta*, *ets1*/*2*, and *tbr*

**Fig. 3:**
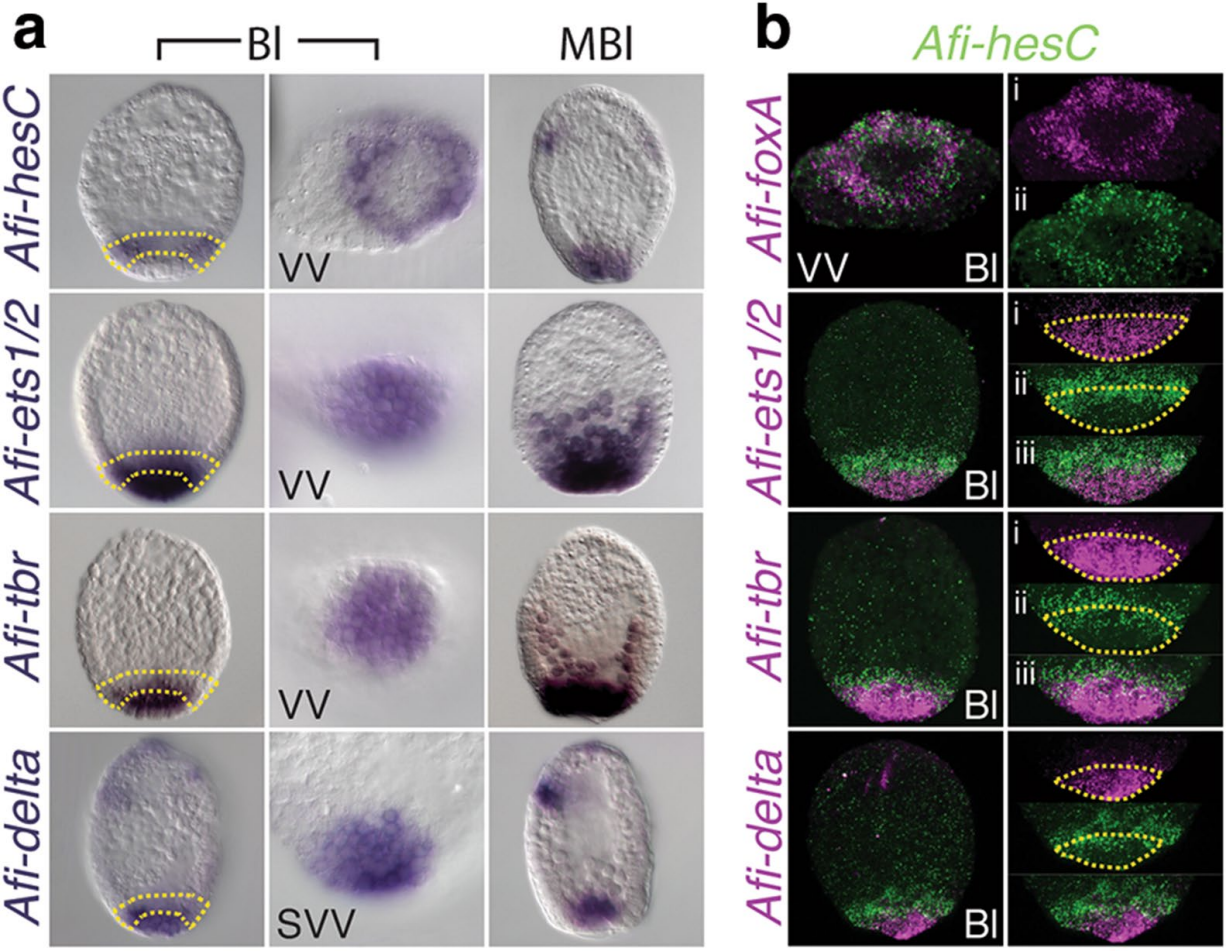
In *A. filiformis* *hesC* is co-expressed with its immediate downstream genes. **a** Single WMISH for blastula and mesenchyme blastula stage embryos. **b** Double fluorescent WMISH on blastula stage embryos. *Afi*-*hesC* expression is restricted to a ring of cells in the vegetal half and co-expressed with the endomesodermal marker *Afi*-*foxA*. *Afi*-*ets1/2*, *Afi*-*tbr,* and *Afi*-*delta* are co-expressed with *Afi*-*hesC* in one cell layer (*yellow area*) at the vegetal plate of the embryo at blastula stage and completely co-expressed at mesenchyme blastula stage. *VV* vegetal view, *SVV* semi vegetal view, *Bl* blastula, *MBl* mesenchyme blastula

In euechinoids, the second element of the DNG consists of the globally expressed gene *hesC*, which is excluded from the skeletogenic lineage by the repressive action of Pmar1. HesC directly represses a cohort of genes encoding for TFs (*ets1*/*2*, *alx1*, *tbr*, and *soxC*) and signaling molecules (*delta*) [2, 38, 42]. This cohort of genes will drive forward the skeletogenic program up to the activation of differentiation gene batteries (Fig. 2d) [2]. On the contrary, in cidaroids a great variability in the expression and function of *hesC* has been recently reported [18, 19]. Although we showed that *Afi*-*pplx* is probably not working as a repressor and thus is not part of a DNG logic, we cannot exclude that *Afi*-*hesC* might still spatially restrict the expression of the same downstream genes via its repressive action. Therefore, we cloned and analyzed the spatio-temporal expression of *Afi*-*hesC* and its immediate downstream genes *Afi*-*ets1/2*, *Afi*-*alx1*, *Afi*-*tbr,* and *Afi*-*delta*. At the aa level, Afi-HesC shows conservation of all its distinctive domains, including the VRPW repressor domain, making it likely to retain a transcriptional repressor function. *Afi*-*hesC* is not detectable throughout cleavage stages (Additional file 1: Figure S5A), and begins to be expressed at blastula stage in a ring of cells towards the vegetal pole (Fig. 3a), thereby acting as a local rather than a global regulator. Importantly, *A. filiformis* orthologs of the DNG downstream genes, *tbr*, *ets1/2*, and *delta*, are partially co-expressed with *Afi*-*hesC* (Fig. 3b) at blastula stage. This co-expression becomes even more extensive at mesenchyme blastula stage, when *Afi*-*hesC* occupies the vegetal plate together with *ets1/2*, *delta,* and *tbr*, making a repressive action of Afi-HesC on these genes highly unlikely (Fig. 3a). Furthermore, double FISH shows complete co-expression of *Afi*-*hesC* with *Afi*-*foxA* at this stage (Fig. 3b), while at mesenchyme blastula stage *Afi*-*hesC* will occupy the center of the vegetal plate delimited by a ring of *Afi*-*foxA* expressing cells (Fig. 3, Additional file 1: Figure S6). In sea urchin, the *Spu*-*foxA* gene is initially expressed in the entire endomesoderm territory apart from the SM lineage, while later in development it gets restricted specifically to the endoderm lineage only [9].

Double FISH shows that *Afi*-*pplx*, *Afi*-*ets1/2*, *Afi*-*tbr,* and *Afi*-*delta* are all expressed in a larger domain than *Afi*-*alx1* (Fig. 3, Additional file 1: Figure S7), a result additionally supported by the counts of positively labeled cells in several embryos. At blastula stage, *Afi*-*ets1/2 is expressed in* 32 (±2) cells (n = 6), *Afi*-*tbr* in 40 (±3) cells (n = 5), and *Afi*-*delta* in 32 (±3) cells (n = 5), which indicates that they are likely expressed in the whole mesoderm and possibly partially in the endoderm territories and are not restricted only to the skeletogenic precursor cells marked by *Afi*-*alx1* 18 (±3) cells (n = 17) as in sea urchin. This is consistent also with the earlier expression of *Afi*-*delta* and *Afi*-*pplx* in a wider domain of 20 (±5) cells (n = 5) compared to 8 (±1) cells (n = 2) of *Afi*-*alx1* at early blastula (Additional file 1: Figure S5). Afi-HesC, however, could still repress *Afi*-*alx1* into a small domain in the center of the vegetal plate of the blastula. Assuming that Afi-HesC is the main direct repressor of *Afi*-*alx1* in the vegetal plate, as shown in euechinoid and in a species of cidaroid sea urchins (*S. purpuratus* [43] and *E. tribuloides* [18]), the expression pattern of those genes should be mutually exclusive. The double FISH identifies some cells that do not express *Afi*-*alx1* within the domain delimited by *Afi*-*hesC,* suggesting either a non-direct relationship between *Afi*-*alx1* and *Afi*-*hesC* or the presence of another repressor of *Afi*-*alx1* in these cells (Additional file 1: Figure S7).

Altogether our analysis suggests the absence of a *pplx/hesC* DNG in brittle star as a mechanism of initial specification of the subdomain of mesoderm expressing skeleton-specific genes and is supported by the following: (1) Partial co-expression at blastula stage and complete co-expression at mesenchyme blastula stage of *Afi*-*hesC* with *Afi*-*tbr*, *Afi*-*ets1/2,* and *Afi*-*delta* support that the cis-regulatory apparatuses of these genes are insensitive to the repression by Afi-HesC and are thus different from euechinoid sea urchin. (2) *Afi*-*pplx* is expressed in 35 (±6) cells (n = 7) similar to *Afi*-*tbr*, *Afi*-*ets1/2,* and *Afi*-*delta* suggesting co-expression with *Afi*-*hesC* and, hence, the absence of a repressive action of *Afi*-*pplx1* on *Afi*-*hesC* at this stage, consistent with the absence of known protein repressive domain. (3) The incomplete mutual exclusive expression of *Afi*-*hesC* with *Afi*-*alx1* makes a role of *Afi*-*hesC* as sole repressor of *Afi*-*alx1* unlikely.

### Other differences between sea urchin and brittle star skeletogenic GRNs

**Fig. 4:**
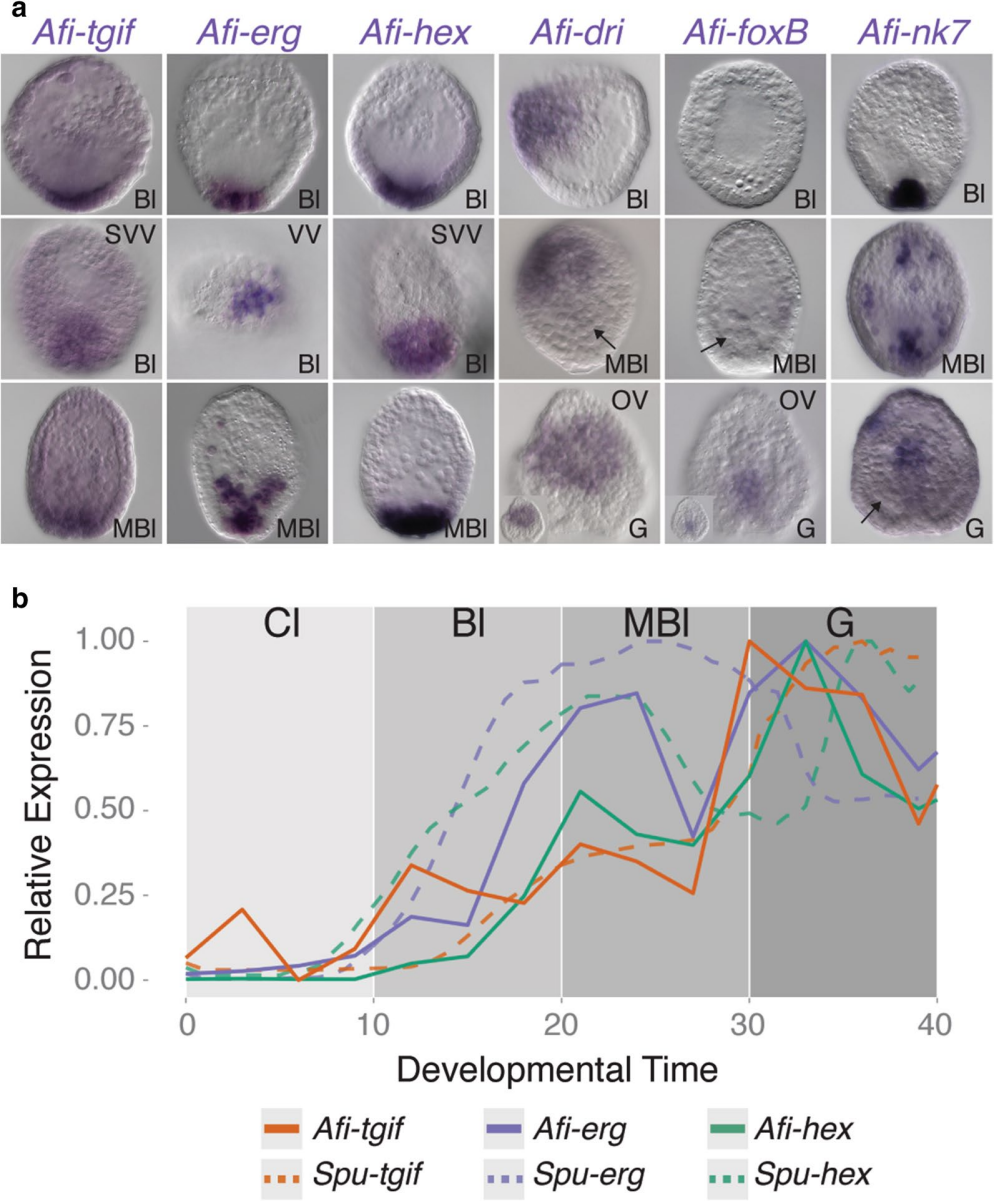
Expression pattern of orthologs of late skeletogenic genes. **a** WMISH showing the expression pattern of of *Afi*-*tgif*, *Afi*-*erg*, *Afi*-*hex*, *Afi*-*dri*, *Afi*-*foxB*, and *Afi*-*nk7* at different developmental stages. At blastula stage, orthologs of the three interlocking loop genes are expressed in the same domain; however, details of their vegetal plates show that *Afi*-*tgif* and *Afi*-*hex* are expressed in a wider domain than *Afi*-*erg*. At mesenchyme blastula, *Afi*-*tgif,* *Afi*-*erg,* and *Afi*-*hex* are co-expressed in NSM cells. From blastula to gastrula, *Afi*-*dri* and *Afi*-*foxB* shows no expression in SM lineage (skeletogenic mesodermal cells shown with *black arrow*). *Afi*-*nk7* shows expression in SM lineage during blastula and mesenchyme blastula stage only. **b** Normalized timeseries comparison shows inverted sequence of onset of expression between brittle star (*solid lines*) and sea urchin (*dashed lines*). Sea urchin data were obtained from [59]. *VV* vegetal view, *SVV* semi vegetal view, *Bl* blastula, *MBl* mesenchyme blastula, *G* gastrula

Downstream of the initial tier of regulation activated by the DNG, the sea urchin SM network is stabilized by an interlocking loop (IL) engaging the genes, *tgif*, *erg,* and *hex* in a recursively wired positive feedback loop [2]. Interestingly, this IL is conserved in mesodermal cells of sea star, an echinoderm class that does not form any larval skeleton [11], suggesting an ancestral function not directly linked to larval skeletogenesis. In *A. filiformis* the genes *Afi*-*tgif*, *Afi*-*erg* and *Afi*-*hex* are expressed or enriched in a group of cells at the vegetal plate of the blastula, similar to sea urchin and sea star, but with the following differences: *Afi*-*erg* is expressed in a smaller domain nested within *Afi*-*hex* expressing cells; *Afi*-*tgif* is ubiquitously expressed at low levels and enriched only in the vegetal plate (Fig. 4a). This is consistent with a transient function of the IL in *Afi*-*erg* positive cells of the vegetal plate only at blastula stage. Whereas *Afi*-*erg* stays active in SM until gastrula stage (Fig. 4a, Additional file 1: Figure S8), *Afi*-*hex* is turned off from SM as soon as these cells enter into the blastocoel (Fig. 4a) and is unlikely to be a driver of skeletogenic genes at later stages. Importantly, at mesenchyme blastula stage these same three genes are now co-expressed in the vegetal plate, where the non-skeletogenic mesodermal cells (NSM) reside, and possibly reestablish the IL in these cells (Fig. 4a). Time-course comparisons between sea urchin and brittle star pinpoint differences of initial inputs responsible for the activation of these genes. In the only echinoderm species where the dynamic of gene expression is available, *S. purpuratus,* the three genes are activated in the following order: *Spu*-*hex*, *Spu*-*erg*, and *Spu*-*tgif*, in all cases needing the former for the activation of its subsequent. Conversely, in brittle star the order of activation is perfectly reverted (Fig. 4b) suggesting differences in initiation and potentially promoter logic of the IL in brittle star compared to sea urchin. It is important to notice that orthologs of the main drivers of the sea urchin and sea star IL genes, Tbr and Ets1/2, in brittle star are expressed not only in SM lineage, but also in a wider mesodermal area consistent with the expression of *hex* and *tgif*, but not *erg*. This implies that *Afi*-*erg* requires extra input(s) to be restricted to a subset of cells at blastula stage. These data are in agreement with an ancient pan-mesodermal role of the *hex*-*erg*-*tgif* IL, as seen in sea star [11], rather than performing a dedicated SM function as evolved in euechinoids.

In sea urchin skeletogenesis, two extra TFs, *foxB* and *dri,* directly regulate the expression of some differentiation genes [44–46]. *Spu*-*foxB* is only employed during larval skeletogenesis in *S. purpuratus*, whereas *Spu*-*dri* is also expressed in an adult skeletogenic domain [21]. In contrast, the brittle star orthologs of these are not involved in larval or adult skeletogenesis ([47] and unpublished data), as confirmed by WMISH and QPCR (Figs. 1b, 4).

Finally, recent transcriptomic screenings in *S. purpuratus* [16, 48] identified three additional transcription factors as specifically expressed in skeletogenic cells during development, although little is known about their role in the GRN for skeletogenesis. These are *Spu*-*nk7*, *Spu*-*alx4*, and *Spu*-*mitf*. From quantitative transcriptome data available in the EchinoBase (http://www.echinobase.org/Echinobase/), it is evident that none of them is expressed before SM ingress into the blastocoel (24 hpf), and spatial data confirm the expression in primary mesenchymal cells only for *Spu*-*nk7* and *Spu*-*mitf* [49, 50]. In sea urchin, *Spu*-*nk7* is expressed in skeletogenic cells from mesenchyme blastula throughout development, suggesting a role in late skeletogenesis. To understand the potential role of these genes in brittle star, we surveyed our transcriptome data; we identified only *Afi*-*nk7* and *Afi*-*alx4*, and analyzed the expression of *Afi*-*nk7*. This gene showed expression already at blastula in the vegetal plate and in the SM cells of the mesenchyme blastula stage, but it is absent from skeletogenic cells at later stages of development and during skeleton deposition (Fig. 4a).

In summary, while the IL might still act in brittle star mesodermal cells, the late skeletogenic regulators, *Afi*-*foxB* and *Afi*-*dri*, are not responsible for driving the expression of any skeletogenic differentiation genes because they are never expressed at the analyzed stages (*mitf*) or never expressed in these cells (*foxb* and *dri*). Additionally, the expression of *Afi*-*nk7* shows heterochronic expression between the two classes of echinoderms.

### Dynamic regulatory states during *A. filiformis* mesoderm development

A recent study showed conservation of the blastula mesodermal regulatory state among different classes of echinoderms, excluding brittle stars [6], although the relative positioning of the different mesodermal cells within the vegetal plate showed a certain degree of variation.

**Fig. 5:**
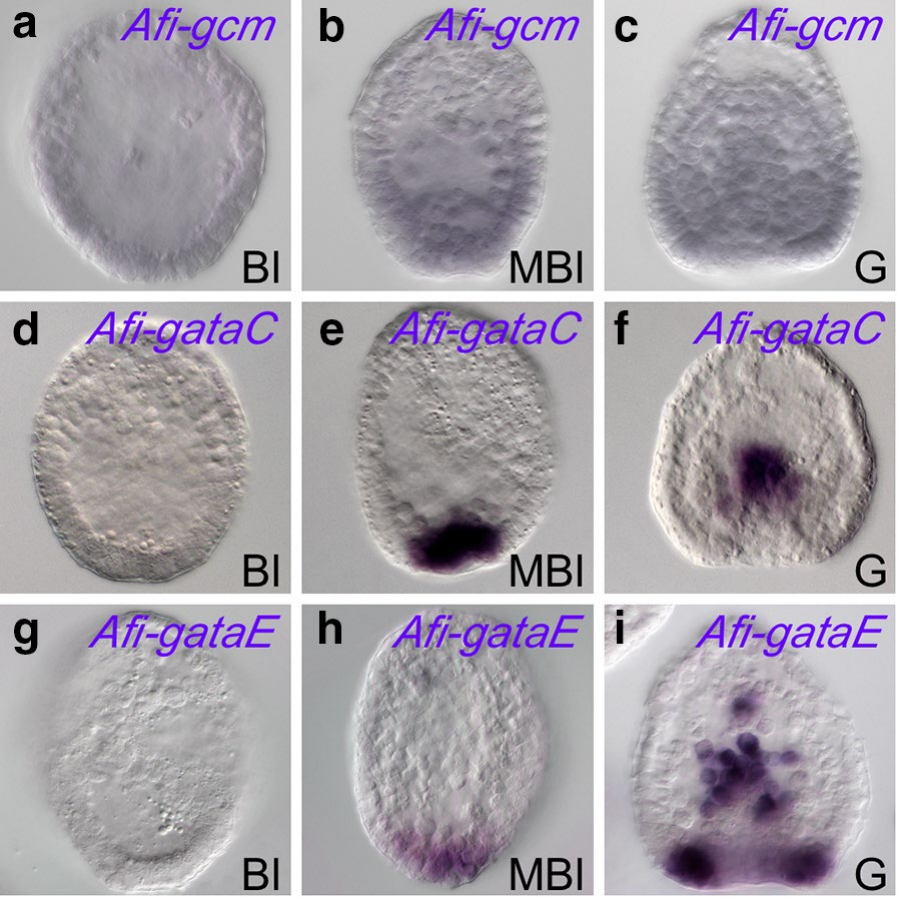
Expression of *A. filiformis* non-skeletogenic mesodermal genes. **a**–**i** WMISH at different developmental stages as indicated in the *bottom right corner* of each image. WMISH probes used are indicated in the *top right corner*. **a**–**c** *Afi*-*gcm* is not detectable by WMISH at any of the stages analyzed, consistent with QPCR expression levels (compare Additional file 1: Table S1). **d**–**f** *Afi*-*gataC* expression becomes detectable at mesenchyme blastula stage in NSM cells in the vegetal plate and it stays active at the tip of the archenteron at gastrula stage. **g**–**i** *Afi*-*gataE* expression becomes active in a similar fashion to *Afi*-*gataC* at mesenchyme blastula stage. At the beginning of gastrulation, it marks the cells at the tip of the archenteron and in the blastopore region. Different to *Afi*-*gataC*, *Afi*-*gataE* is additionally expressed in cells at the base of the archenteron in the blastopore region. Both *Afi*-*gataC*, *Afi*-*gataE* are not expressed at blastula stage. *Bl* blastula, *MBl* mesenchyme blastula, *G* gastrula

To understand the timing of specification and the disposition of various mesodermal cells (*i.e.*, skeletogenic and non-skeletogenic mesoderm) within the vegetal half of the embryo, we performed a series of in situ hybridizations on NSM regulatory genes, using *Afi*-*alx1* as a landmark for SM and *Afi*-*foxA* for its outer boundary (Fig. 5, Additional file 1: Figure S6 and S7). We found no expression of NSM specification genes (*gataE*, *gataC,* and *gcm*) during blastula stage in *A. filiformis* (Fig. 5). At this stage, the SM is eccentric to the boundary delimited by *Afi*-*foxA/Afi*-*hesC*, establishing, thus, a third small mesodermal domain of unknown function (Additional file 1: Figure S7B and C). A few hours later, at mesenchymal blastula, once the SM cells ingress into the blastocoel, *Afi*-*gataE* and *Afi*-*gataC* are expressed in the entire vegetal plate (Fig. 5) along with *Afi*-*hesC* (Additional file 1: Figure S7) and other mesodermal genes (*i.e.,tbr*, *ets1*/*2*, *tgif*, *erg,hex*, and *delta*). In sea urchin, Alx1 represses the NSM driver gene *gcm* in the SM lineage to ensure spatial separation of these two types of mesoderm. The absence of expression of *Afi*-*gcm* in brittle star (confirmed both with WMISH Fig. 5, and QPCR, Fig. 1b) makes this network linkage unlikely to exist. The late expression of *Afi*-*gataE* and *Afi*-*gataC* also suggests that NSM specification and patterning might occur at later stages compared to sea urchin.

**Fig. 6:**
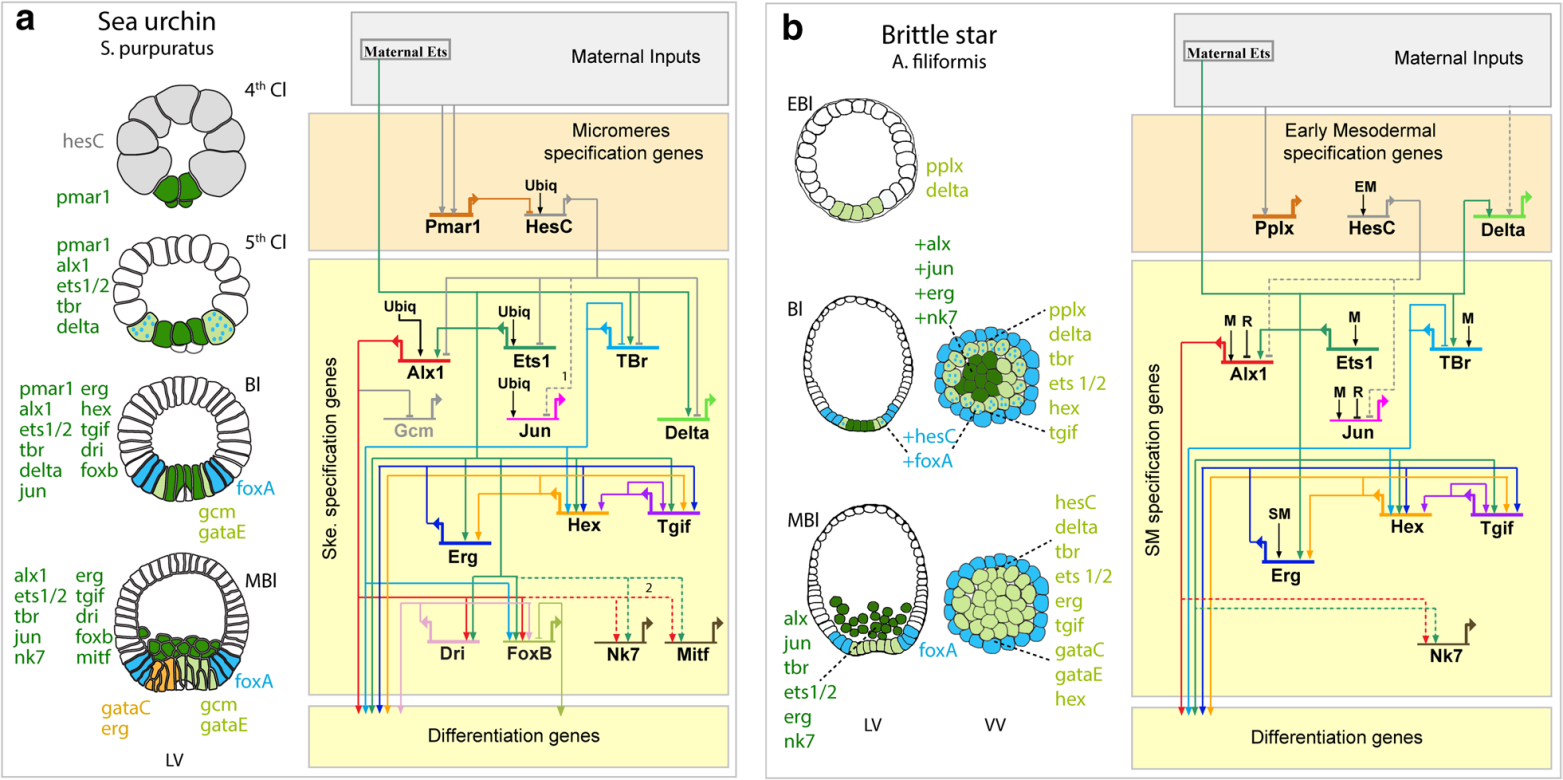
Summary and comparison of regulatory states and minimal GRN rewiring between *A. filiformis* and sea urchin. **a** Regulatory states (*left*) and GRN model (*right*) for *S. purpuratus*. Regulatory states for the mesodermal territories are shown on cellular maps of cleavage (Cl), pre-hatching blastula (EBl), hatched blastula (Bl) and mesenchyme blastula (MBl). The *dark green* represents the skeletogenic mesoderm (SM), the *light green* represents the non-skeletogenic mesoderm (NSM), and the blue represents the endodermal territory. At mesenchyme blastula, the NSM is divided into precursors of pigment cells (*orange*) and other NSM precursors (*light green*). Genes expressed in each territory are listed. *S. purpuratus* SM-GRN architecture, modified from [2], of the genes analyzed in this study. Arrows indicate positive inputs (activation) and barred line negative inputs (repression). Dashed lines represent functional linkages inferred by perturbation data in [2] for 1 and [16] for 2. Ubq represents an inferred ubiquitous activator necessary for the expression of some of the genes downstream of the DNG. **b** Regulatory states (*left*) and potentially conserved GRN linkages (*right*) for *A. filiformis*. Regulatory states for the mesodermal territories are shown on cellular maps of pre-hatching blastula (EBl), hatched blastula (Bl), and mesenchyme blastula (MBl). The *light green* cells represent the mesoderm identified by the expression of pan-mesodermal genes. The *dark green* represents the here named SM cells expressing the skeletogenic marker genes *alx1*, *jun,* *p58a, p58b,* and *p19;* blue dots represent the expression of *hesC* and *foxA* within the mesoderm territory, while blue cells represent the endodermal territory that surrounds the mesoderm. At blastula stage, the SM is clearly separated from the rest of NSM, while the segregation of NSM and endoderm occurs at MBl stage. Genes expressed in each territory are listed. A hypothetical *A. filiformis* GRN including nodes and potentially conserved linkages represented as in (**a**) and based on the observations in this study. EM represents the minimal hypothetical positive input necessary for the *hesC* expression in the endomesoderm. M represents hypothetical pan-mesodermal positive input(s) necessary to drive the expression in all mesodermal cells. R represents hypothetical repressor(s) expressed in cells expressing only pan-mesodermal genes. SM represents the minimal hypothetical positive input necessary for *erg* to be expressed in the subset of mesodermal cells. This hypothetical model highlights the differences (nodes and linkages) based on the expression data. As discussed in the text, we parsimoniously assume that, in absence of functional data, linkages are conserved with sea urchin, but we cannot exclude alternative hypotheses

Cell specification is not a single-step process and several genes contribute to different aspects of this biological process emphasizing the importance of studying the dynamics of regulatory states. Therefore, we built a cellular resolution map (Fig. 6b) of the different mesodermal regulatory states up to mesenchyme blastula stage integrating all presented data. Our analysis revealed that (1) only *Afi*-*pplx* and *Afi*-*delta* have localized expression in a group of cells already visible by the end of cleavage stage (Fig. 2, Additional file 1: Figure S5). (2) Most of the mesodermal genes, including the SM genes, start their zygotic expression around early blastula stage (12 hpf), suggesting that the initiation of mesoderm specification might occur at this stage (Fig. 1b). (3) After hatching, a cohort of regulatory genes is expressed in all mesodermal cells and likely specifies a pan-mesodermal state. These are *Afi*-*tbr*, *Afi*-*ets1/2,Afi*-*tgif*, *Afi*-*hex*, *Afi*-*pplx*, and *Afi*-*delta*. (4) At this stage, a unique combination of transcription factors characterizes at least three distinct mesodermal domains (Fig. 6b, light green). An SM domain marked by the expression of *Afi*-*alx1*, *Afi*-*jun, Afi*-*nk7*, and *Afi*-*erg* (Fig. 6b, dark green). A small domain expressing only the pan-mesodermal genes *Afi*-*tbr*, *Afi*-*ets1/2,Afi*-*tgif*, *Afi*-*hex*, *Afi*-*pplx,* and *Afi*-*delta* (Fig. 6b, light green), and lastly, a one cell-wide ring of overlap between *Afi*-*foxA* and *Afi*-*hesC* and the pan-mesodermal genes (Fig. 6b, light green with blue dots). Additionally, expression of *Afi*-*foxA* and *Afi*-*hesC* spans towards the presumptive endoderm (Fig. 6b, blue). (5) By mesenchyme blastula, the SM and the NSM are now completely segregated although both express pan-mesodermal genes. The SM is now composed of mesenchymal cells, which have ingressed into the blastocoel and express *Afi*-*alx1, Afi*-*nk7*, and *Afi*-*jun* as distinctive markers. The NSM remains in the vegetal plate of the blastula epithelium and is distinguished by the expression of *Afi*-*hesC*, *Afi*-*delta*, *Afi*-*hex*, *Afi*-*tgif*, *Afi*-*gataC,* and *Afi*-*gataE*.

## Discussion

Understanding the molecular basis of evolutionary changes to GRNs resulting in the gain or loss of specific complex characters is a fundamental question in biology. The depth of knowledge of the molecular mechanisms for endomesoderm specification in different echinoderm classes [7, 8, 11] provides an exceptional opportunity to unravel differences and similarities in GRN architecture responsible for evolution of cell type and complex morphological structures. In this study, we fill an important gap left open in echinoderm comparative GRN studies, by analyzing the dynamic of regulatory states taking place during the development of brittle star mesoderm.

In the pluteus larvae of sea urchins and brittle stars, the elaborated mineralized skeleton is formed by a sub-population of mesodermal cells specifically expressing the transcription factors *alx1* and *jun*. Our developmental observations in *A. filiformis* show that these skeletogenic cells ingress as mesenchymal cells into the blastocoel before gastrulation, similarly to what has been already described for another ophiuroid species (*Ophiopholis aculeata*; [28]) and to euechinoid, rather than cidaroid sea urchins. Despite the morphological similarities of the skeleton and its mode of development in both echinoderm classes, several differences are revealed by our high-resolution multi-gene expression analysis (summarized in Fig. 6), which have important implication in GRN architecture. Foremost, side-by-side comparison of brittle star and sea urchin mesodermal regulatory states (Fig. 6) reveals that many differences in network architecture are apparent at the level of the initiation of the expression of skeletogenic genes, and at the level of the regulation of the differentiation gene batteries. This is consistent with the “hourglass” model of evolution [51, 52] in which evolutionary plasticity of network linkages differs depending on their position in the network hierarchy (early, mid or late). Our analysis also shows that gene duplication, protein function diversification, and *cis*-regulatory evolution took place over 480 million years of evolution to shape the larval skeletogenic networks as we see them now in the echinoderm classes which develop pluteus-like larvae.

### Evolution of GRN by protein function diversification

Skeletogenesis is the most studied developmental program in echinoderms including both sub-classes of sea urchin and for this reason provides an exceptional opportunity for multi-element comparison. In euechinoids, the most similar developing embryo to *A. filiformis*, the skeletogenic program is unlocked at cleavage stage by the action of the *pmar1/hesC* DNG. A potential ortholog of the sea urchin Pmar1 has been identified in *A. filiformis* (*Afi*-*pplx)* and in another brittle star species [30]; however, our cross-species analysis revealed functional non-equivalence of these transcription factor genes. Important gene duplication and protein function diversification events likely occurred to shape the sea urchin Pmar1 into a potent repressor of the DNG. Our protein phylogeny (Fig. 2a, Additional file 1: Figure S3) supports a likely ancient duplication of the *phb1* gene, which led to the emergence of the *pplx/pmar1* class in the last common ancestor of sea urchins and brittle stars, ~480 Mya. The relaxed selective pressure on the new duplicated *pplx/pmar1* gene may have then allowed the diversification of the protein function by the acquisition and loss of short linear protein motifs (e.g., eh1), which are reported to be easily evolvable [41]. The presence in all euechinoid species analyzed so far of several almost identical copies of *pmar1* (Fig. 2a) arrayed in tandem in the same fragment of DNA (Sea Urchin Genome Project BAC Clones #170H13 and #020N20; GeneBank AC131562) suggests a further gene duplication event that possibly occurred concomitant with the emergence of the stereotypical quartet of micromeres present at the vegetal pole of the euechinoid embryo. The ancient duplication hypothesis is further supported by the striking similarity of spatio-temporal expression pattern between the *Spu*-*pmar1* and the *Afi*-*pplx* (Fig. 2) gene, both expressed transiently at the vegetal pole of the early embryo.

The *pmar1/pplx* gene type has so far been identified only in euechinoids and ophiuroids, possibly due to limited sequence resources available for other echinoderm species. Most publicly available data consist of transcriptome data of specific developmental stage(s) and do not sample early embryonic stages, when the *pmar1/pplx* gene is likely to be expressed (Fig. 2c). An exception is given by the draft of the sea star *P. miniata* genome [53], for which we can state confidently that no *pmar1/pplx* gene is present, although a clear *phb1* gene has been identified (Fig. 2a). Our evolutionary scenario in light of recent echinoderm phylogeny [22–25] implies the loss of the *pmar1/pplx* gene at least in the Asteroidea lineage, although we cannot exclude the alternative hypothesis of independent duplication of *pmar1* and *pplx* genes from a *phb1* ancestor gene, respectively, in the Echinoidea and Ophiuroidea lineages. Both scenarios, however, pinpoint the high evolutionary plasticity of the *pmar1*/*pplx* gene and its role in the GRN underlying echinoderm mesoderm specification.

### Evolution of GRN by changes in *cis*-regulation

High-resolution expression data of *Afi*-*hesC* and its immediate targets (*Afi*-*ets1/2*, *Afi*-*tbr*, *Afi*-*delta*) integrated with the echinoderm phylogeny allow the reconstructing of the nature and timing of the molecular changes occurred to set up the DNG responsible for the precocious specification of skeletogenic fate in micromeres in the euechinoid lineage (Fig. 3b). As laid out in Fig. 6, the predicted changes in *cis*-regulatory apparatus of *ets1*/*2*, *delta*, and *tbr* to become negatively regulated by HesC, and for the *hesC* gene to become repressed by Pmar1 [21], likely occurred in the euechinoid lineage once split from the cidaroid sister group. Support for this is given by the pencil urchin (cidaroids) *hesC*, the expression of which is not consistent with a function in a DNG at the top of the skeletogenic regulatory cascade [11, 18, 19]. In cidaroids as well as in asteroids [11] and ophiuroids (this study), *hesC* is co-expressed with its sea urchin immediate targets, *ets1*/*2*, *tbr*, and *delta*, which are expressed in all mesodermal cells and not only restricted to the skeletogenic lineage as in sea urchin (Fig. 5b). This implies that *ets1*/*2*, *tbr,* and *delta* in *A. filiformis*, as well as other classes, cannot be directly repressed by HesC and that a different mechanism must exist to drive their precise spatial expression pattern. A possible molecular mechanism emerged recently in sea star, where expression of these genes was shown to be regulated by sustained high levels of nuclear β-catenin [54]. It is interesting to notice that the expression pattern of *hesC* is dramatically different in the species so far studied (and belonging to different classes) ranging from a ubiquitous expression excluded only from SM (euechinoids), or excluded from the vegetal ectoderm (asteroids and *Prinocidaris baculosa* cidaroid) to a restricted ring of cells in the vegetal half of the embryo (ophiuroids and *E. tribuloides* cidaroid) highlighting high evolutionary plasticity of the *hesCcis*-regulatory apparatus.

Other substantial differences between the sea urchin and the brittle star are identified at the periphery of the skeletogenic network, at the level of the downstream differentiation genes, which in *A. filiformis* are not regulated by the TF Afi-FoxB and Afi-Dri either in the larva or in the adult ([47] and unpublished data). While *foxB* expression only in skeletogenic cells of euechinoid embryos supports a co-option of this gene in the larval skeletogenesis of echinoids, the absence of *dri* function in the brittle star skeleton GRN is specific to ophiuroids, given the fact that in both sea urchin and sea star *dri* is expressed in association with skeletogenic cells [21]. Extensive rewiring at the level of *cis*-regulatory apparatus of several differentiation genes as well as *foxb* and *dri* themselves must have occurred accordingly to change the expression in the two classes of echinoderms.

### Common regulatory state for skeletogenesis

The precise spatio-temporal expression of a developmental gene is controlled by its own *cis*-regulatory apparatus, which is capable of integrating multiple inputs (i.e., transcription factors) present in a given combination (regulatory state). Therefore, if no changes occur in the inputs or in the *cis*-regulatory apparatus of a developmental gene during evolution, the expression pattern of orthologous genes will be maintained in related species. While our experimental approach cannot demonstrate the existence of linkages, it can certainly exclude their existence. Therefore, from the comparison of different spatial or temporal expression of orthologous genes in the two species, we can infer regulatory differences in the skeletogenic GRN of sea urchin and brittle star, given an identical regulatory state. Conversely, we presume, in the absence of perturbation data in *A. filiformis*, that when similar expression patterns are observed, the regulatory functional connections are conserved with sea urchin embryos that produce skeleton. For instance, we cannot address if the *alx1* gene is regulating skeletogenic genes, as it does in sea urchin, or if it regulates other mesodermal genes as in sea star early embryo. Therefore, we might actually underestimate the amount of architectural differences between the two networks in the two classes of echinoderms. Figure 6b highlights the differences of network linkages, based on the high-resolution expression data here reported, compared to the *S. purpuratus* GRN model (Fig. 6a, [2]). Although, functional studies are needed for validation, expression of *alx1* and *jun* at blastula stage, along with the pan-mesodermal regulatory genes *ets1*/*2, tbr, tgif, hex, and erg,* seems to be a common feature of the initial state of the skeletogenic program in both euechinoid and brittle star, as well as in sea cucumbers [6], which produce small mineralized spicules. This set of regulatory genes likely constitutes the right combinatorial code necessary to drive echinoderm larval skeletogenesis. A direct consequence of this hypothesis is that many of the downstream genes should be directly regulated by Alx1 and other mesodermal TFs in a combinatorial fashion, and thus be expressed long before the mineralized skeleton is formed. Indeed, in sea urchin as well as in brittle star (Fig. 1) several mineralization genes are expressed already at blastula stage, and genome wide analyses identify *alx1* and *ets1/2* as major controllers of these differentiation genes [16, 17]. Furthermore, these two transcription factors are expressed in the plesiomorphic adult skeleton of brittle star, sea star, and sea urchin [21, 47].

### Independent or common evolution of larval skeleton?

Our data reveal substantial differences in the regulatory programs underlying the development of mineralized skeleton in the morphologically similar pluteus larvae of sea urchins and brittle stars. Adult calcitic endoskeleton is a plesiomorphic character of echinoderms apparent also in ancient extinct species [55]. It has been proposed that the sea urchin larval skeleton evolved by a simple co-option of an ancient adult skeletogenic GRN module [21], consistent with the expression of many skeletogenic regulatory gene also in adult skeleton, as already discussed. What about the ophiuroid larval skeleton? Did it originate with the same co-option event of sea urchin or with an independent co-option event? To address this question, two key aspects need to be considered: (1) the phylogenetic relationship of the five echinoderm classes; (2) the developmental specific network nodes rather than the regulatory genes expressed also in adults and being part of an ancient skeletal GRN module.

The recent availability of transcriptomic and genomic data from several echinoderm species sprung several phylogenomic analyses based on large dataset including representative of all echinoderm extant classes [22–25]. All these extensive molecular phylogenies converge on the consensus tree, in which echinoderms and holothurians form a distinct clade (Echinozoa) from the ophiuroids and asteroids clade (Asterozoa) and the crinoids are sister taxa to these four classes (Eleutherozoa). These new studies bring back the divergence between Echinozoa and Asterozoa roughly 480 Mya [23].

If the larval skeleton originated independently, the co-option of the adult skeletogenesis GRN module would have occurred during brittle star larval evolution using different developmental genes compared to sea urchin. In fact, many sea urchin-specific skeletogenic developmental genes, such as *foxb*, *dri,* and *mitf* are not expressed in cells (or not expressed at all) that also express skeleton matrix genes in *A. filiformis*, and therefore, it is conceivable that they are not part of the larval skeletogenic GRN. On the other hand, in the case of a single co-option event (common evolution), the role of at least some developmental only genes should have been conserved (e.g., *pmar1*, *hesC,* and *foxB*) between brittle star and sea urchin. In this case, the larval skeleton would have originated only once at the base of the Eleutherozoa and would have been lost in asteroids and reduced in holothurians. Despite all, the ancient split (roughly 480 Mya) of all classes and their long branches of independent evolution make a clear conclusion difficult. Importantly, regardless of common or independent evolution, it is likely that the co-option happened through the same genes *alx1* and *jun* and thus at the same tier of the GRN in both echinoderm classes. This implies that the *cis*-regulatory control initiating the expression of *alx1,* and possibly *jun,* in a subset of mesodermal cells should reveal the exact evolutionary nature of sea urchin and brittle star co-option event and it would be a prime focus for future investigations.

## Conclusion

In conclusion, assuming that the subset of mesodermal cells is indeed skeletogenic, our study establishes *A. filiformis* as a key developmental system for a detailed comparative analysis of the gene regulatory networks acting in the skeletogenic mesoderm in distantly related echinoderms. Our high-resolution gene expression study identifies differences and similarities in the mesodermal regulatory states between *A. filiformis* and euechinoids. These have important implications for the GRNs underlying mesoderm in brittle stars and set up a clear framework for future functional experiments in *A. filiformis.* Independently of this, our work brings new evidence to a long debated issue on the evolutionary origin of echinoderm larval skeleton and clarifies specific mechanisms of GRN diversification, which see not only *cis*-regulatory elements evolution, but also gene duplication and protein function diversification as equally important mechanisms.

## Methods

Embryo cultures were set up as previously described in [56]. cDNA synthesis and QPCR for high-resolution time-courses were done as described in [47] using *Afi*-*16S* as internal standard. WMISH were performed as described in [57] with the following changes: pre-hatching embryos were treated with 1 mg/ml Trypsin (Sigma) for two hours before fixation; hybridization temperature of 55 °C. FISH were performed as described in [58], using the hybridization buffer described in [57] and hybridization temperature of 55 °C. Cell counts were estimated using confocal z-stacks with the Cell Counter plugin part of Fiji/ImageJ on several embryos. Microinjections were performed as described in [40]. All synthetic mRNAs are described in Additional file 1: Figure S4; and they were injected at equimolar concentration, for detailed procedures see Additional file 2.

## Additional files

10.1186/s13227-015-0039-x Additional figures and tables; Contains figures and tables supporting data in the main text.
10.1186/s13227-015-0039-x Extended methods; Contains a detailed description of methods used to conduct study.

## Abbreviations

GRN: gene regulatory networks
Mya: million years ago
DNG: double negative gate
TF: transcription factor
WMISH: whole mount in situ hybridization
QPCR: quantitative polymerase chain reaction
FISH: fluorescent in situ hybridisation
SM: skeletogenic mesoderm
HD: homeodomain
Afi: *Amphiura filiformis*
Pmi: *Patiria miniata*
Spu: *Strongyloncentrotus purpuratus*
aa: amino acid
GFP: green fluorescent protein
IL: interlocking loop
NSM: non-skeletogenic mesoderm
ECl: early cleavage
Cl: cleavage
EBl: early blastula
Bl: blastula
MBI: mesenchyme blastula
LMBl: late mesenchyme blastula
G: gastrula
LG: late gastrula
VV: vegetal view
SVV: semi vegetal view
