# Supplementary material for: Large-scale gene expression study in the ophiuroid Amphiura filiformis provides insights into evolution of gene regulatory networks
Source: EvoDevo. 2016 Jan 11;7:2. doi: 10.1186/s13227-015-0039-x (PMC4709884; doi:10.1186/s13227-015-0039-x)
Supplement: Supplementary file 1 — 10.1186/s13227-015-0039-x Additional figures and tables; Contains figures and tables supporting data in the main text. [file 13227_2015_39_MOESM1_ESM.pdf]

SUPPLEMENTARY FIGURES

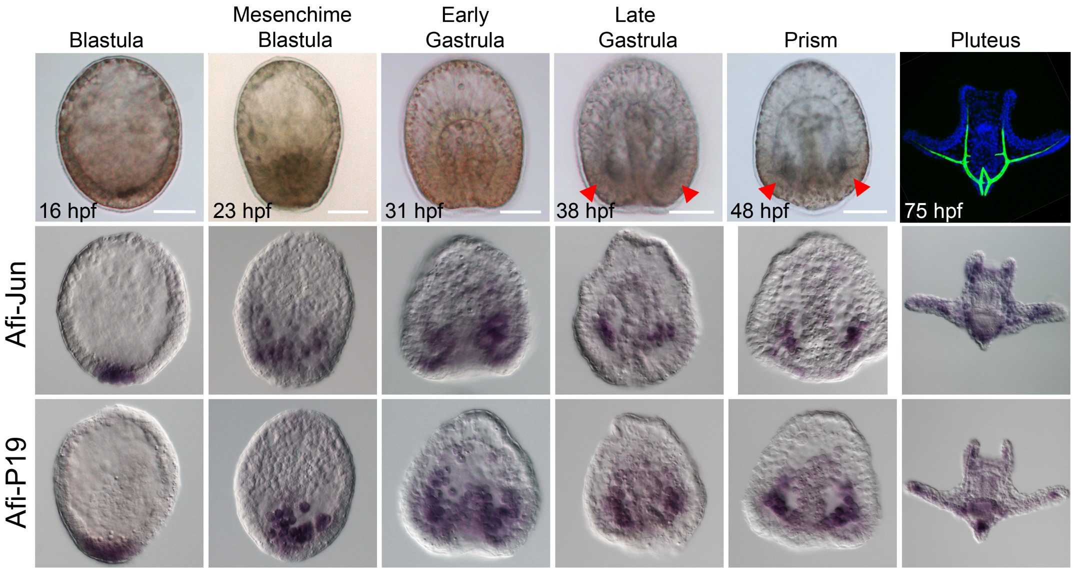

Figure Supplement 1. *Afi-jun* and *Afi-p19* are expressed in skeletogenic cells throughout development. First row shows light microscopy images of *A. filiformis* embryos at different developmental stages: from blastula onwards. The two bio-mineralized spicules become clearly visible in the blastocoel of the gastrula associated with clusters of mesencymal cells. Later tetra-radiated spicules extend to a full skeleton as visible in the calcein stained (green) embryo at pluteus stage (75hpf). Second and third row show single colorimetric whole mount in situ hybridization (WMISH) of *Afi-jun* and *Afi-p19* in *A. filiformis* developmental stages as indicated above. Both genes are initially expressed in a group of cells in the vegetal plate, then in the first ingressing cells of the mesenchyme blastula stage and then in two groups of cells at the base of the archenteron throughout gastrulation. At pluteus stage the expression of Afi-jun and Afi-P19 mirror the elaborated skeletal structure. Images for blastula, mesenchyme blastula and late gastrula stage of *Afi-p19* are the same as in Fig 1. Expression of these genes is always visible where the skeletal elements are formed.

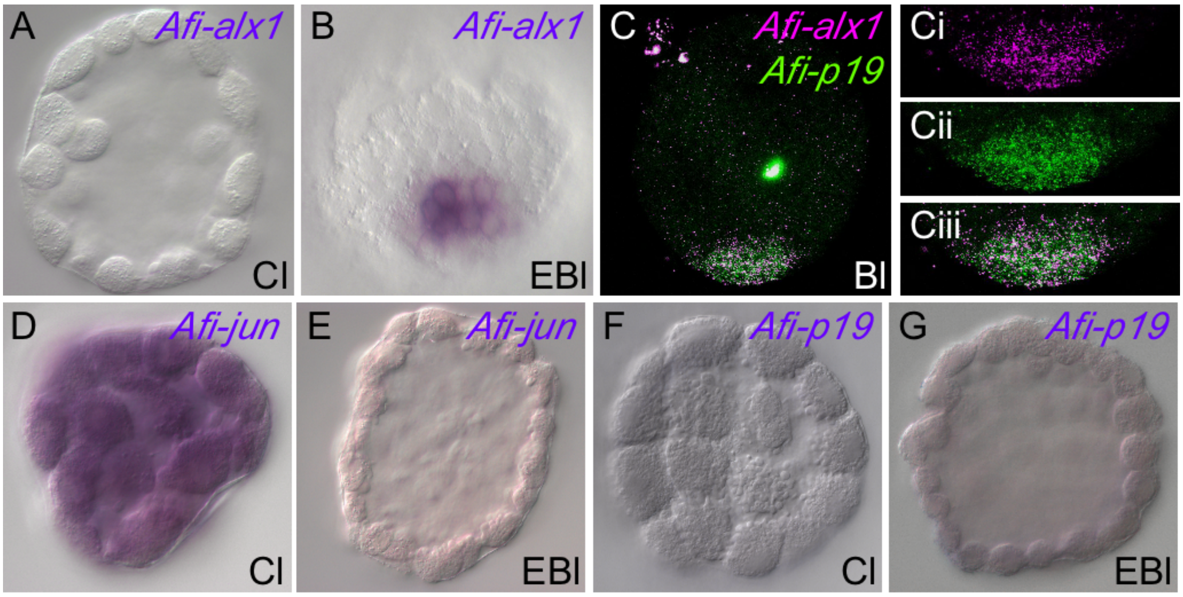

Figure Supplement 2. Skeletogenic precursor cells are specified at blastula stage. (A-G) Single colorimetric and double fluorescent whole mount in situ hybridization (WMISH) in *A. filiformis* early developmental stages. (A and B) Consistent with the quantitative data, the expression of *Afi-alx1* is not evident at late cleavage, but becomes detectable in 8 (±1) cells at early blastula stage. (C) Double FISH shows that *Afi-alx1* and *Afi-p19* are completely co-expressed at blastula stage. (D-E) WMISH of *Afi-jun* in very early stages of development shows that the maternal expression, still present at cleavage stages, is ubiquitously distributed and not yet restricted to the SM cells. (F-G) Although the quantitative analysis of *Afi-p19* transcript abundance shows expression of this gene at early pre-blastula stages, it is not yet restricted to a subset of cells. Cl – cleavage, EBl – early blastula, Bl - blastula.

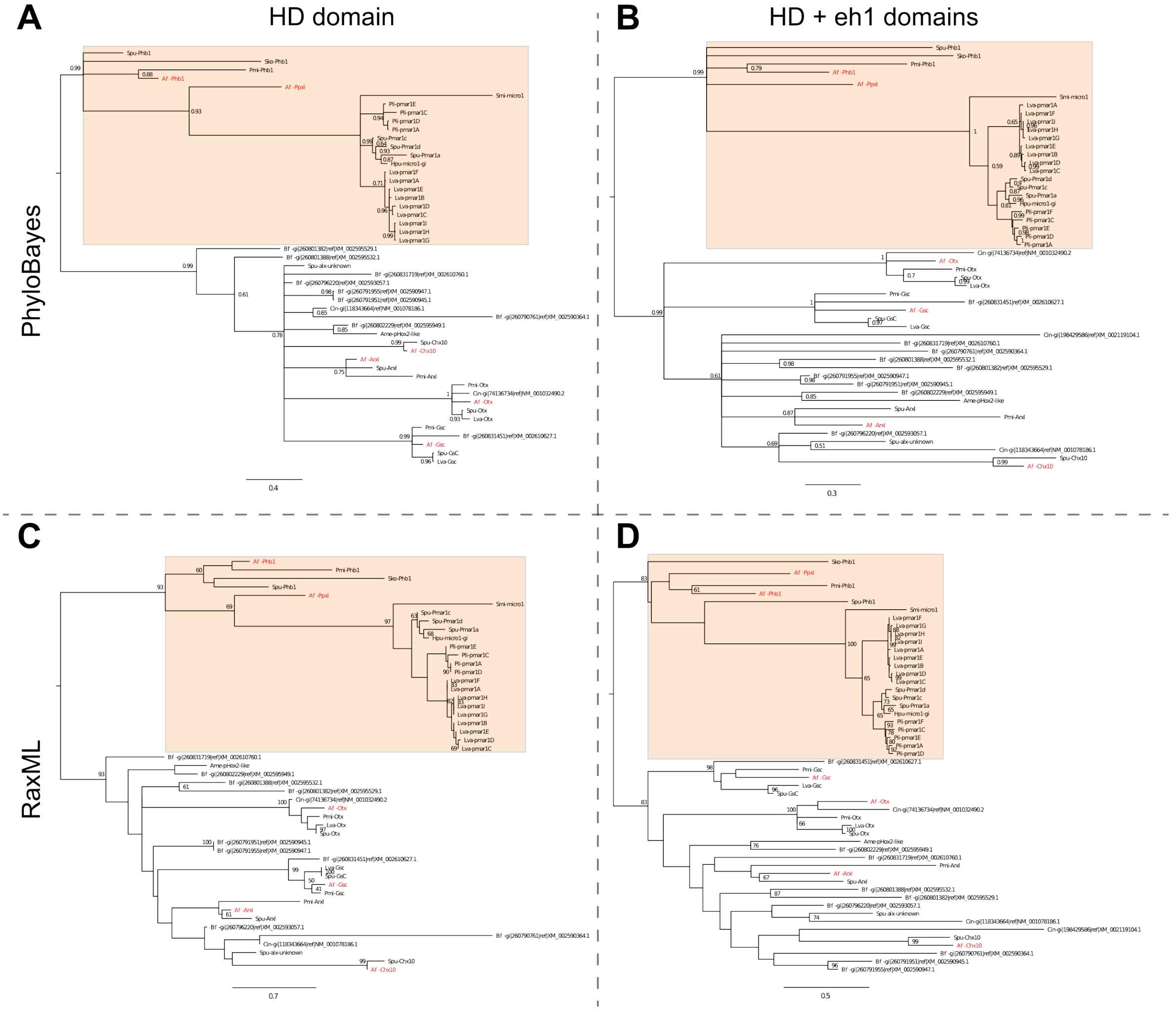

Figure Supplement 3. Phylogenetic trees of two different alignments with two different methods support grouping of Afi-Pplx with Phb1 and Pmar1 genes. (A) Posterior consensus tree estimated using only the HD as alignment shows orthology of Afi-Pplx with Pmar1 supported by a posterior probability of 0.93. The grouping of Afi-Pplx and Pmar1 as sistergroup to Phb1 genes is supported with a posterior probability of 0.99. (B) Posterior consensus tree estimated using the HD with additional two eh1 repressor domains supports a polychotomy of Afi-Pplx with Phb1 and Pmar1 genes with a posterior probability of 0.99. (C) Maximum likelihood tree estimated using 10,000 bootstraps of only the HD as alignment supports orthology of Afi-Pplx with Pmar1 genes with a bootstrap of 69. The split of Afi-Pplx and Pmar1 genes from the Phb1 genes is supported with a bootstrap of 93. (D) Maximum likelihood tree estimated using 10,000 bootstraps of an alignment of HD with additional two eh1 repressor domains supports independent duplication of Afi-Pplx from Afi-Phb1 and Spu-Pmar1 from Spu-Phb1, however with low bootstrap values, *i.e.* below 60. On the other hand grouping of Pplx with Phb1 and Pmar1 genes is supported with a bootstrap of 83. (A-D) In the pink boxes is the pplx gene with the phb1 and pmar1 genes. Outside of the pink box are various closely related HD paired like genes obtained through a blast search of sea urchin Pmar1 amino acid sequence against various echinoderm and chordate databases. In all four trees this grouping is consistent. All trees are rooted to their midpoint and only values are shown above 0.6 for the bayesian trees and 60 for the maximum likelihood trees.

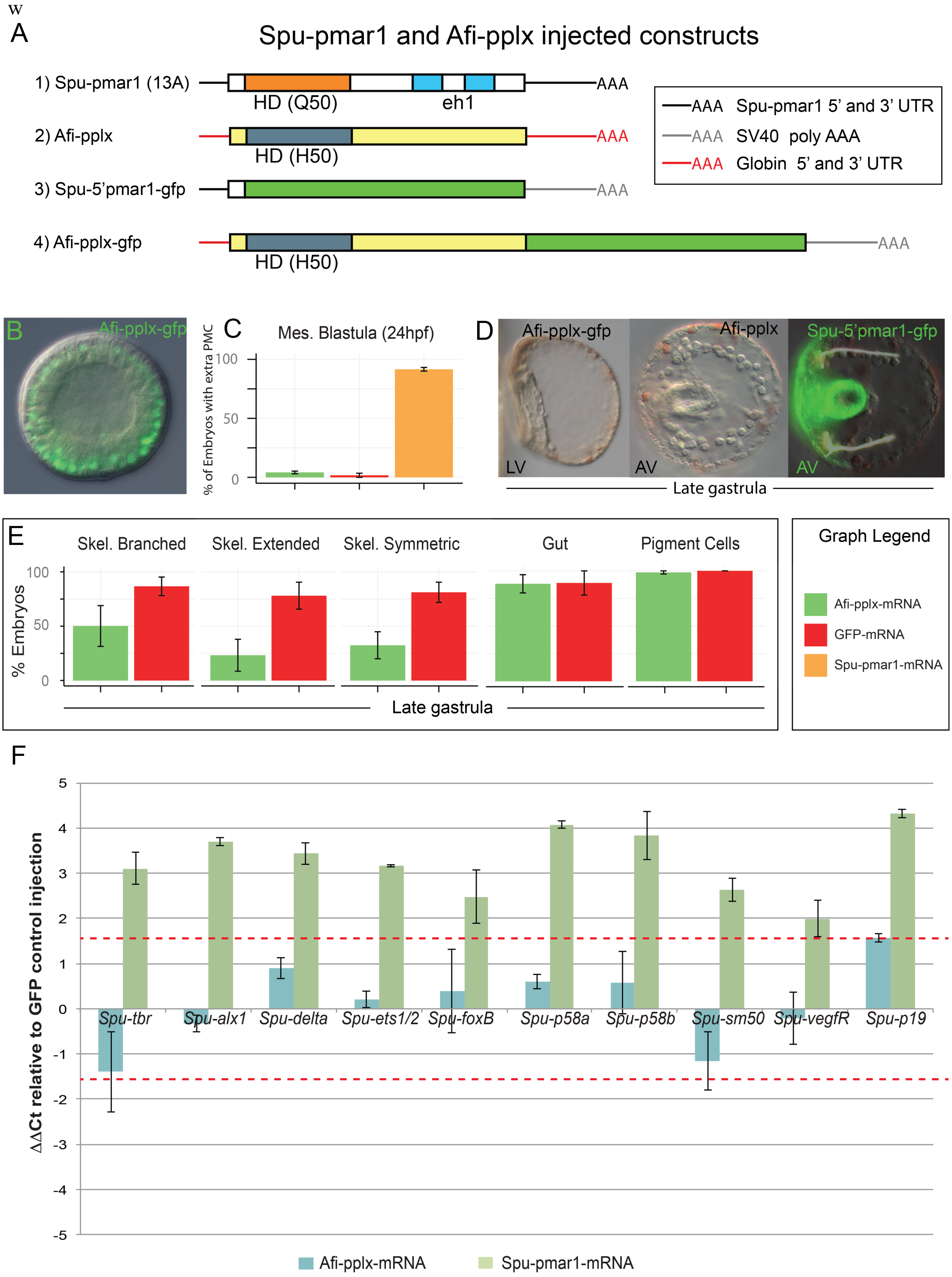

Figure Supplement 4. *Afi-pplx* is functionally different from the sea urchin *pmar1* repressor. (A) Schematics of constructs used for micro-injection of sea urchin fertilised eggs. (B) Afi-pplx-gfp is successfully translated and shows GFP expression in nuclei of sea urchin embryos at 18hpf. (C) Injection statistics obtained at 24hpf comparing the percentage of embryos with elevated PMC count. Afi-pplx injected sea urchin embryos do

not induce all cells to become PMCs as observed in the ectopic expression of endogenous Spu-pmar1 in sea urchin embryos. For Afi-pplx-mRNA 38/34, GFP-mRNA 16/21 and for Spu-pmar1-mRNA 50/39 embryos were checked for phenotype (replicate 1/replicate2). (D) Phenotypical observations on living embryos shows for Afi-pplx-gfp and Afi-pplx injections a reduction of skeleton. No such reduction is observed in embryos injected with Spu-5'pmar1-gfp at 48hpf. (E) Injection statistics showing percentage of embryos with effect on different morphological features at 48hpf (late gastrula). Consistent with the phenotype in D, Afi-pplx injected embryos show effects in branching, extension and symmetry of the larval skeleton in sea urchin. No effect on the other hand is observed in gut formation and abundance of pigmented cells. For Aif-pplx-mRNA 33/39 and for GFP-mRNA 30/32 embryos were checked for phenotype (replicate 1/replicate2). The graphs indicate the percentage of embryos at 48 hpf scored for fully extended gut (Gut); presence of pigment cells (Pigment); tri-radiate branched spicules (Skel. Branched); extension of skeleton similar to controls (Ske. Extended); and presence of two symmetric skeletal elements (Skel. Symmetric). Error bar represents standard deviation of two independent biological replicas. (F) QPCR on injected embryos for sea urchin skeletogenic genes. The graph represents the normalized cycle of difference ($\Delta\Delta$Ct) relative to GFP control injection. The values are calculated as described in [1]. For Spu-pmar1-mRNA all tested genes show a significant increase in transcript level. For Afi-pplx-mRNA no such increase is observed. Error bars represent standard deviation of 4 technical replicas.

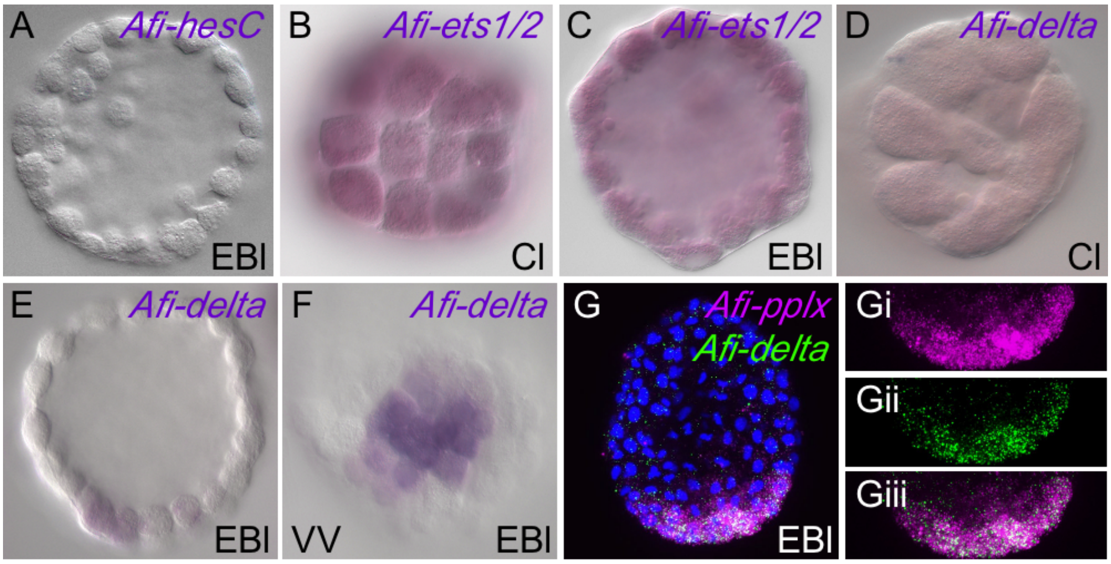

Figure Supplement 5. Expression of mesodermal genes in early stages of *A. filiformis* development identifies only *Afi-delta* localised at early blastula stage in the same cells as *Afi-pplx*. (A-G) Single and double fluorescent WMISH. *Afi-hesC* (A) is not visible at cleavage stage, while *Afi-ets1/2* (B-C) is ubiquitously expressed at cleavage and early blastula stage. (D-F) *Afi-delta* spatial expression in early embryo stages. Although, quantitative data (Table S1) show low levels of expression of *Afi-delta,* WMISH (D) fail to detect a localised expression before early blastula stage when it gets restricted to 19 (±6) cells. (G) Double WMISH showing *Afi-delta* and *Afi-pplx* co-expressed at early blastula stage. Developmental stages are indicated at the bottom right corner of each image and probes at the top tight. Cl - cleavage; EBl - early blastula; VV - vegetal view.

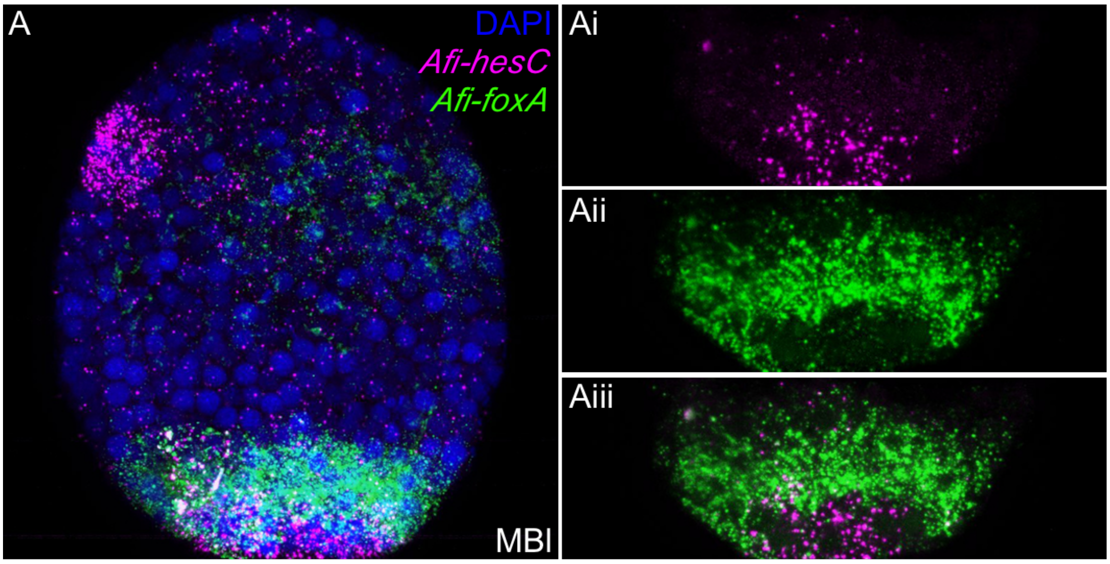

Figure Supplement 6. Expression of *Afi-hesC* and *Afi-foxA* at mesenchyme blastula stage (MBl) show no co-expression. (A) Double fluorescent *in situ* of *Afi-hesC* and *Afi-foxA. Afi-hesC* (Ai) is expressed at the center of the vegetal plate in NSM cells and in a small domain in the ectoderm. While, *Afi-foxA* (Aii) marks the endoderm, which is a ring surrounding the *Afi-hesC* domain. (Aiii) Maximum projection of 3 z-stack slices shows mostly no co-expression of the two genes. MBl - mesenchyme blastula.

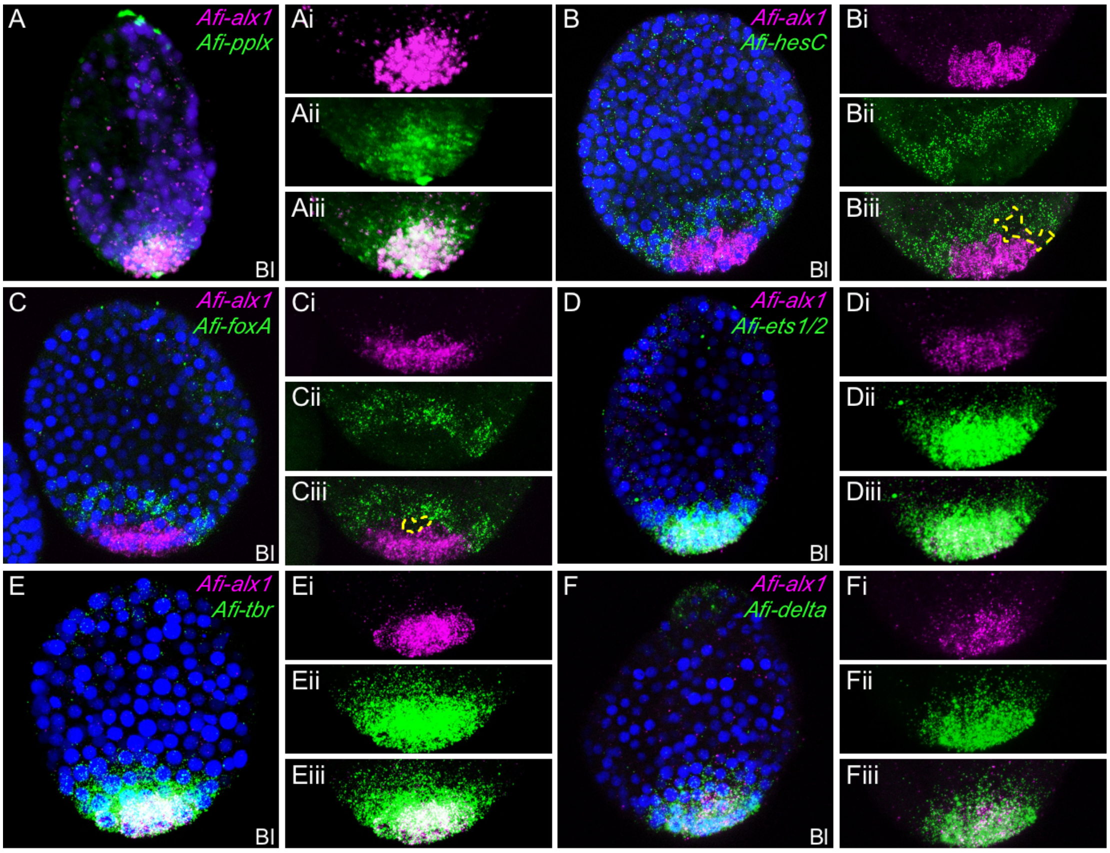

Figure Supplement 7. The endomesodermal regulatory states of the brittle star *A. filiformis* at blastula stage. (A-F) Double FISH using *Afi-alx1* expression as marker for SM cells. (A) *Afi-pplx* is expressed in a wider domain than *Afi-alx1*. (B) *Afi-hesC* is expressed as ring around the vegetal pole of the embryo and is surrounding *Afi-alx1*. The area in yellow shows cells in the vegetal plate that do not express neither *Afi-hesC* nor *Afi-alx1*.(C) Expression of *Afi-foxA* and *Afi-alx1* during blastula stage is similar to (B) and consistent to the complete co-expression of *Afi-hesC* and *Afi-foxA* at this stage (Fig 4). (D-F) *Afi-ets1/2, Afi-tbr* and *Afi-delta* are expressed in a large domain in the vegetal plate larger than *Afi-alx1*. Cell nuclei are stained with DAPI (blue). Bl – blastula

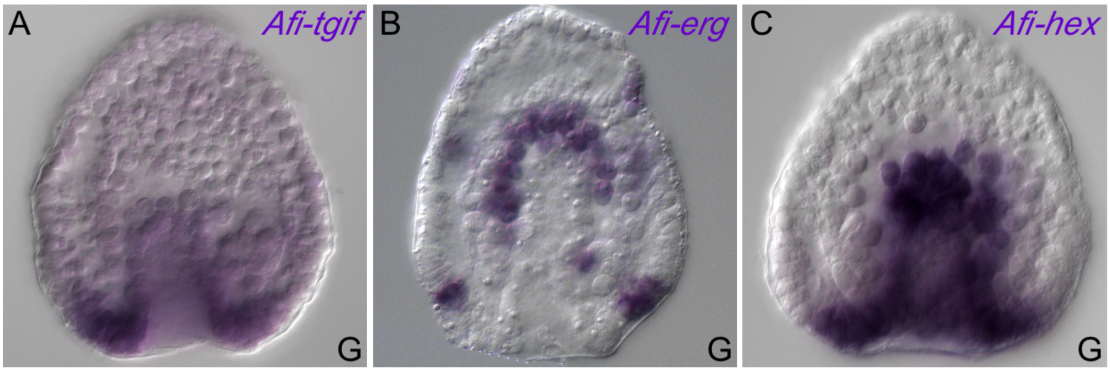

Figure Supplement 8. At gastrula stage none of the feed-back positive loop genes is any longer expressed in SM cells. (A) *Afi-tgif* marks the endomesoderm of the gastrulating embryo. (B) *Afi-erg* shows scattered expression in other non-skeletogenic mesenchymal cells. (C) *Afi-hex* is expressed in many endodermal and mesodermal cells, but absent from the skeletogenic domain. G – gastrula.

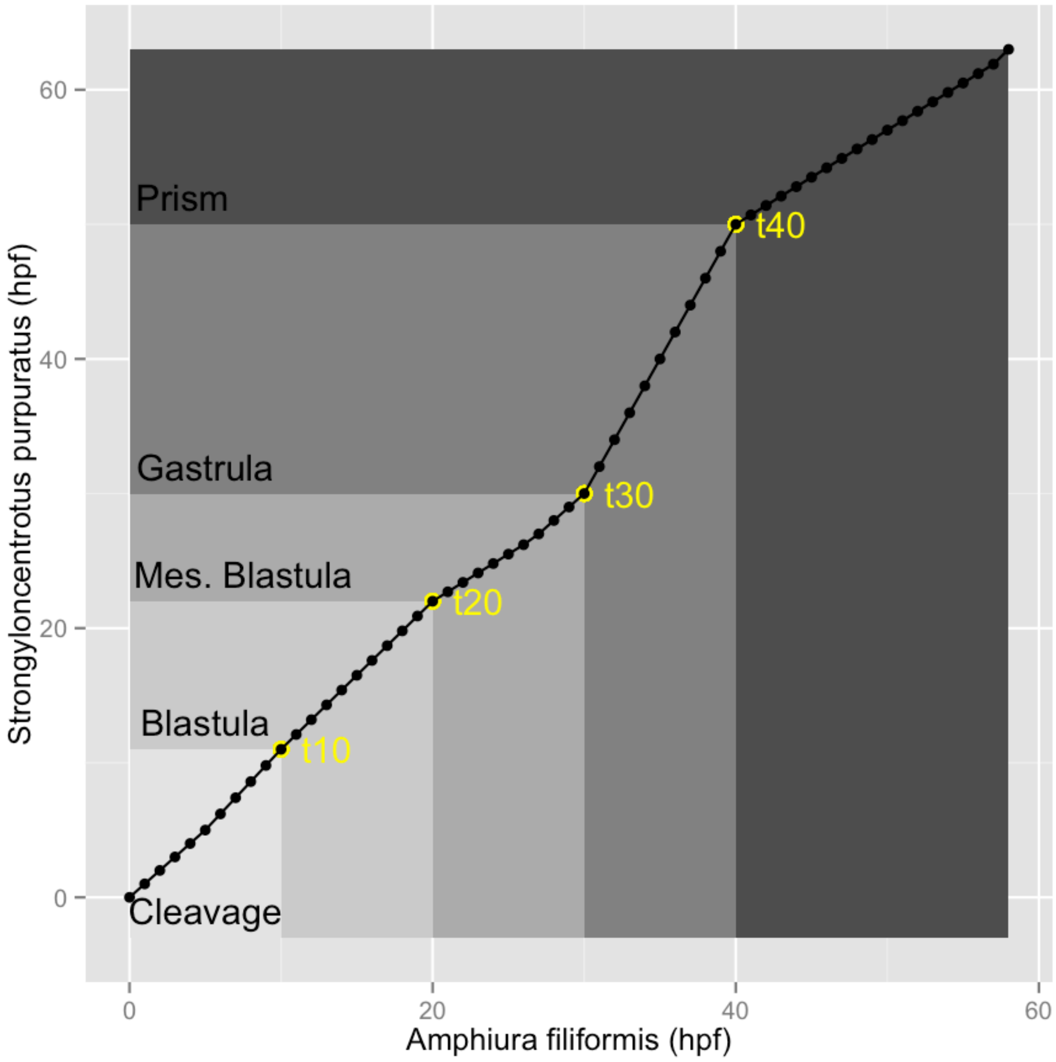

Figure Supplement 9. Normalization of developmental timing according to stages between *Amphiura filiformis* and *Strongyloncentrotus purpuratus*. This normalization allows plotting of time-course data of sea urchin and brittle star according to their individual stages of development in standard laboratory conditions (Fig. 2 and Fig. 4).

SUPPLEMENTARY TABLES

Table S1. Time-courses for endo-mesodermal genes in *Amphiura filiformis*

Afi-alx1	0	0	0	3	63	67	101	165	141	100	79	122	88	68	60
Afi-p19	0	0	0	4	66	199	479	1084	907	1216	1578	2281	2078	1603	1597
Afi-jun	1143	830	1614	487	690	476	1072	1993	1370	695	1789	1385	992	1204	1866
Afi-pplx	9	11	50	233	482	206	143	127	22	15	43	27	19	13	17
Afi-hesC	44	15	4	3	21	12	36	42	41	27	76	168	NA	NA	NA
Afi-ets12	1686	3396	3215	2455	2250	979	1130	1972	1887	1694	2128	5158	3466	2882	3392
Afi-tbr	36	43	33	14	48	132	305	805	993	673	1447	1292	NA	NA	NA
Afi-delta	75	95	64	72	313	265	209	201	148	148	204	429	343	214	250
Afi-tgif	89	283	0	125	461	359	309	545	476	348	1358	1170	1145	627	1098
Afi-erg	14	20	31	53	138	120	428	592	625	312	625	737	614	458	573
Afi-hex	5	7	4	4	75	108	379	852	659	610	923	1531	930	775	894
Afi-dri	1	1	1	0	0	5	19	26	34	28	25	21	49	51	39
Afi-foxB	0	0	0	0	0	1	2	37	12	15	81	145	174	124	220
Afi-gcm	3	5	6	27	24	7	12	7	11	13	21	15	11	7	22
Afi-gataC	0	0	1	1	1	4	3	24	47	67	105	145	82	52	40
Afi-gataE	1	12	12	7	17	13	23	117	81	157	376	740	1130	861	1057
Afi-foxA	1	6	8	4	11	41	233	980	1152	790	2315	3888	3043	2914	4248
**Gene**	**0**	**3**	**6**	**9**	**12**	**15**	**18**	**21**	**24**	**27**	**30**	**33**	**36**	**39**	**42**

Developmental time (hpf)

Table Supplement 1. High resolution time-courses for every 3hr of development up to 42hpf obtained by QPCR. Values shown were normalized using Afi-16S and multiplied by 1,000,000

Table S2. Cloning primers and clone lengths

Gene	Or.	Cloning	Length	GenBank
Afi-Alx1	F	Czarkwiani et al 2013	634bp	KC788414
	R			
Afi-Jun	5O	ACCATGGACGGATCAAACAT	429bp	KM816839
	5I	GCCATTTAGCTCTGCGATTT		
Afi-P19	3O	TCGCATAGGTCTTGGGAAAC	697bp	KM816840
	3I	CCCTCCAACAGACCAAGAAA		
Afi-PPLX	F	GCTTCGTGAGAAAGCGATGC	799bp	KM816841
	R	TAGCTTGGCAAGTTCACGGG		
Afi-PPLX	F	GCTCCAACGAGAGCGAA	948	
	R	TCAAAACTTGGGTACACTGCAGATG<br>G		
Afi-HesC	3O	TGTTTCCTGGAAGCTGTGTG	1414bp	KM816842
	3I	CATTGTCTTTGCCCTTGTT		
Afi-Delta	F	TGCAACGGATCAGGCTCAAT	2156bp	KM816843
	R	GGCGATGAGTCCGGTGTATT		
Afi-Ets1/2	F	Czarkwiani et al 2013	671bp	KC788415
	R			
Afi-Tbr	F	Czarkwiani et al 2013	1127bp	KC788418
	R			
Afi-Erg	F	GCGCATCGTGGTCAAATACC	2149bp	KM816844
	R	GCTTGACGCAACTTGGGAAG		
Afi-Hex	F	TTGTCAAGTGGGCAGTTCGT	1243bp	KM816845
	R	CTTTGGCACAACAGCACTGG		
Afi-Tgif	F	TCGCCAAAGCTAGCTGTCAA	1305bp	KM816846
	R	CCGAGTCTGACTTCAGCTTCAT		
Afi-Dri	5O	GTCTTCCCTCACGACGATTG	1482bp	KM816847
	5I	CTCACGACGATTGCCATCTA		
Afi-FoxB	F	Czarkwiani et al 2013	424bp	KC788416
	R			
Afi-GataC	F	Czarkwiani et al 2013	1021bp	KC788417
	R			
Afi-GataE	F	TCAATTCAACTGGAGATAGCGC	1736bp	KM816848
	R	GAGAGTTGCCGATGTGTGTC		
Afi-Gcm	F	TCTCTGCGACAAATCCTGCC	1156bp	KM816849
	R	CTCATCGCGCTTGTATTGCT		
Afi-Phb1	F	CAACCAACCAAAGCTGAGCC	910bp	KM816850
	R	TGGAGGAGCAGATGTCTTGG		
Afi-FoxA	F	CCGGCTGAGTTCTCGAGTTT	1788bp	KM816851
	R	TTGTTTGCGCTGTGGCTAAC		

Table S3: Primers used for QPCR

Gene	Orientation	Primer	Length
Afi-alx1	F	CCAAGTGGAGGAAACGAGAA	157bp
	R	GCTGGTGGTTGTGTGATGTC	
Afi-jun	F	CGGACGTACAAATGCTGAAA	171bp
	R	AGCTGCCTGTTCTTCGGTAA	
Afi-p19	F	CCCTCCAACAGACCAAGAAA	120bp
	R	CCTCTTGCTTCCTTCAGTGAG	
Afi-pplx	F	TCTGCCTTTGCTGACAACCA	168bp
	R	TGACGACGTCTCACTTGTGG	
Afi-hesC	F	AATCAGGTAGCGGCTCAAAC	146bp
	R	GGCCAGGTGGTTCAAGATT	
Afi-delta	F	GCGAAACACTCGATCACTGC	169bp
	R	TCTGAGATGCACGTTCCACC	
Afi-ets1/2	F	CGCGGCTAAGTTTCTCGTAG	133bp
	R	AACCTGCCAACACATCATCA	
Afi-tbr	F	TGATCCCAACCAGTGGAAGT	184bp
	R	CCATTGTCTTTGCCCTTGTT	
Afi-erg	F	CAACAGCAGCAAGGAAACGG	151bp
	R	GTTGCACACTTTCGTGTCCG	
Afi-hex	F	CAGGTCCGGTTCTCAAACGA	159bp
	R	CCTCCATTTCGCCCTTCTGT	
Afi-tgif	F	CGGTCCATCCATGTTTCCGTA	128bp
	R	TGTTTGGCGAGTAGCGATGA	
Afi-dri	F	ACCGCAGTAGTCAGCGGTAT	144bp
	R	TGCAGATAATGCATGGGTGT	
Afi-foxB	F	ACTTGAAACGCTTTCGTCGT	130bp
	R	GCAGAACTCTTTGCGTCACA	
Afi-gataC	F	GACCGCGTGGTTATAAGGAG	131bp
	R	ACTGAATGGCGGGTGTGT	
Afi-gataE	F	TCAAACCACAAAGACGGCTG	135bp
	R	TGAAGTAGAGACCGCAAGCA	
Afi-gcm	F	CAGCTAAACATGCACTGCTGG	133bp
	R	TCACGCAATTGTCTGGCAAC	
Afi-foxA	F	GGCTATGAAGCAATGGCAGC	145bp
	R	TTGTTTGCGCTGTGGCTAAC	
Afi-16S	F	CGGCTGCAGTACTCTGACTG	150bp
	R	GGGTCTTCTCGTCCCACTCT	

REFERENCES

1. Oliveri P, Davidson EH: **Gene regulatory network controlling embryonic specification in the sea urchin**. *Current Opinion in Genetics and Development* 2004:351–360.
