# Supplementary material for: Large-scale gene expression study in the ophiuroid Amphiura filiformis provides insights into evolution of gene regulatory networks
Source: EvoDevo. 2016 Jan 11;7:2. doi: 10.1186/s13227-015-0039-x (PMC4709884; doi:10.1186/s13227-015-0039-x)
Supplement: Supplementary file 2 — 10.1186/s13227-015-0039-x Extended methods; Contains a detailed description of methods used to conduct study. [file 13227_2015_39_MOESM2_ESM.docx]

# EXTENDED METHODS

## Animal Collection and Embryo Culture

Adult animals of *Amphiura filiformis* were collected during their reproductive season (July-August) in the Gullmarsfjord, Sweden. For usage the animals were sieved from the mud and kept in buckets under constant seawater flow and a temperature between 12-15^o^C. *A. filiformis* embryo culture was set up as previously described [1].

## Embryo Collection and RNA Extraction

Total RNA samples were extracted from embryos of different developmental stages. A desired number of embryos (1000E to 10.000E) were re-suspended in at least 10 volumes of RLT (Qiagen). For each RNA extraction we used 1000E applying the RNAqueous Micro kit (Ambion, Life Technologies). Since the embryos were stored in RLT, firstly 1 volume of lysis buffer was added, and then incubated at 65 ^o^C for 10min. After this step the rest of the RNA extraction followed the manufacturer’s protocol. For every sample DNAse treatment was carried out as described in the manufacturer’s protocol. The equivalent of 1000 embryos or 2 μg of total RNA was reverse transcribed using the iScript kit (BioRad) accordingly to manufacturer instructions. The final cDNA was diluted to the equivalent of 2 embryos or 2.7ng per μl.

## Quantitative PCR

For the high-resolution time course data in Amphiura filiformis, RNA was extracted from samples collected every 3hr. QPCR reactions were set up as described in [2]. For each reaction the equivalent of one embryo was used as template and for each gene at least three technical replicas were performed on a 384 well plate. Primer-pairs for each gene (Additional file 1: Table S3) were designed using the PRIMER3 software with the following parameters (Product size: 110-150bp, Max Poly-X: 3, Max 3’ Stability: 8) [3]. Determination of a suitable housekeeping gene was performed using the BestKeeper analysis [4] and the gene *Afi-16S* was selected. The abundance of *Afi-16S* transcripts per embryo was estimated using the Taq-Man Cells-to-CT Control Kit (Ambion, Life Technologies) as spike. In the lysate corresponding to 1000 embryos 1,000,000 xeno-RNA molecules (equivalent of 1000 transcripts per embryo) were introduced. Extraction was performed as described above. Based on replicas on 35 stages (0-72hpf, every 3hr), the correlation value between the xeno-RNA and the *Afi-16S* was estimated to 0.85 with an estimate of average abundance being ~25,000,000/embryo. Fold change was calculated by normalization against *Afi-16S* and amplification efficiency of 1.9 and for simplicity multiplied by 1,000,000. For *Strongyloncentrotus purpuratus* RNA extraction has been performed as for *A. filiformis* and QPCR as described in [6] with primers collected from www.echinobase.org.

## Embryo Collection and Fixation

Pre-hatched embryos were treated for two hours with 1 mg/ml Trypsin (Sigma) in FSW to soften the fertilization membrane. All embryos of different developmental stages were fixed in a solution containing 4% PFA, 32.5% FSW, 32.5mM MOPS pH7, 162.5mM NaCl for at least one hour at 4^o^C. The fixation step was followed by 3 washes in MOPS buffer (0.1M MOPS pH7, 0.5M NaCl and 0.1% Tween-20) on ice or at 4 ^o^C. The embryos were stored indefinitely in 80% EtOH at -20 ^o^C.

## Whole Mount in Situ Hybridization (WMISH)

### Enzymatic WMISH

Embryos stored in 80% EtOH were first rehydrated using 70%, 50% and 25% EtOH washes. In order to get the embryos used for the upcoming treatment 4-5 washes with TBST (0.2M Tris pH7.5, 0.15M NaCl, 0.1% Tween-20) were applied. This was followed with a 1:1 wash in TBST and fresh hybridisation buffer (HB; 50% De-ionized formamide, 10% PEG, 0.6M NaCl, 0.02M Tris pH7.5, 0.5mg/ml yeast RNA, 0.1%Tween-20, 5mM EDTA, 1X Denhardst). The embryos were then pre-hybridised in HB for 1 hour at 55 ^o^C without probe. To a fresh HB each probe was added at a final concentration of 0.05 ng/μl and hybridised overnight at 55 ^o^C. Excess probe was washed with 1X of 75%, 50% and 25% of HB in TBST, then 2 washes in TBST and finally 2 washes in 1X SSC and 1 wash in 0.1X SSC, all at 55 ^o^C. For the antibody detection the embryos were incubated in blocking buffer (BB; TBST with 5% Goat Serum) containing either Anti-Dig AP (Roche) or Anti-DNP AP (TSA) at a dilution 1:1000 for at least 1 hour. Excess of antibody was removed with 5 TBST washes at room temperature and the chromogenic reaction was conducted using NBT/BCIP (Roche) in AP buffer (AP; 0.1M Tris pH9.5, 0.1M NaCl, 0.05M MgCl2, 0.1% Tween-20, 1mM Levamisole) with 10% dimethylformamide. The embryos were stored in 30% or 50% Glycerol and imaged using the Zeiss AxioZoom M1 light microscope.

### Multi-Colour Fluorescent WMISH

The multi-colour fluorescent WMISH protocol follows generally an adapted version of a protocol used in [7] with the following changes. All the washes were performed using MABT (0.1M Maleic Acid pH7.5, 0.15M NaCl, 0.1% Tween-20). The hybridisation was done for at least the 2 days at 55 ^o^C. The hybridisation buffer and probe concentration used was the same as for the enzymatic WMISH. All antibodies were conjugated with horseradish peroxidase and both TSA Anti-DNP- HRP (Perkin Elmer) and TSA Anti-DIG-POD (Perkin Elmer) were applied at a concentration of 1:1000 in the blocking buffer provided by the TSA kit (0.5% blocking buffer in MABT). The embryos were stained with TSA-Cy3 and TSA-Cy5 (Perkin Elmer) for at least 15min. A nuclear counter staining with 1 μg/ml of DAPI (Roche) was done at the moment of observation. Embryos were imaged using the either the Zeiss 510 or the Leica SPE2 confocal microscope. All images were post-processed using Fiji/ImageJ.

## Injection of synthetic mRNA in sea urchin zygotes.

The *Afi-pplx* sequence was cloned adding a synthetic stop-codon just after the conserved coding sequence identified in several isoforms present in the *A. filiformis* developmental transcriptome. Similar to the *Spu-pmar1* expression construct previously described [6] the *Afi-pplx* coding sequence was cloned into a vector containing the Xenopus β-globin 5’ and 3’ UTR [8]. Capped synthetic RNA for injections was synthesized with Sp6 mMessage machine (Life Technologies). To control for successful translation the *Afi-pplx* sequence was also fused with GFP and injected (*Afi-pplx-GFP*). Injection were performed as in [6] using the same controls at equimolar concentrations. The *Afi-pplx* transcript was injected at the final concentration of 11ng/μl in the injection solution, while the Afi-pplx-GFP was injected at the final concentration of 66 ng/μl in the injection solution. The other mRNAs where injected at the same concentration as already published in [8]. Injections were performed in two independent batches. Cloning primers can be found in the Additional file 1: Table S2.

## Phylogenetic analyses

For the phylogenetic tree we selected several amino acid sequences identified using BLAST search of Spu-Pmar1c and Spu-Phb1 on various echinoderms and non-vertebrate deuterostome databases. Initial alignment was performed using MAFFT [9] with standard parameters. Afterwards, empty regions were manually trimmed in order to obtain either only the homeodomain sequence (HD) or the homeodomain with additional two engrailed repressor domains (HD + eh1) part of the Pmar1 sequence. Both alignments were used as starting point for phylogenetic tree reconstruction. Phylogenetic trees were estimated using RaxML v8.0.14 [10] with 10,000 bootstraps, the LG model with Gamma distribution and PhyloBayes [11] using a CAT-GTR model until the maxdiff parameter converged below 0.015.

# REFERENCES

1. Dupont S, Thorndyke W, Thorndyke MC, Burke RD: **Neural development of the brittlestar Amphiura filiformis.** *Dev Genes Evol* 2009, **219**:159–166.

2. Czarkwiani A, Dylus D V, Oliveri P: **Expression of skeletogenic genes during arm regeneration in the brittle star Amphiura filiformis.** *Gene Expr Patterns* 2013, **13**:464–472.

3. Rozen S, Skaletsky H: **Primer3 on the WWW for general users and for biologist programmers.** *Methods Mol Biol* 2000, **132**:365–386.

4. Pfaffl MW, Tichopad A, Prgomet C, Neuvians TP: **Determination of stable housekeeping genes, differentially regulated target genes and sample integrity: BestKeeper – Excel-based tool using pair-wise correlations**. *Biotechnol Lett* 2004, **26**:509–515.

5. Oliveri P, Tu Q, Davidson EH: **Global regulatory logic for specification of an embryonic cell lineage.** *Proc Natl Acad Sci U S A* 2008, **105**:5955–5962.

6. Oliveri P, Carrick DM, Davidson EH: **A regulatory gene network that directs micromere specification in the sea urchin embryo**. *Dev Biol* 2002, **246**:209–228.

7. Andrikou C, Iovene E, Rizzo F, Oliveri P, Arnone MI: **Myogenesis in the sea urchin embryo: the molecular fingerprint of the myoblast precursors.** *Evodevo* 2013, **4**:33.

8. Lemaire P, Garrett N, Gurdon JB: **Expression cloning of Siamois, a Xenopus homeobox gene expressed in dorsal-vegetal cells of blastulae and able to induce a complete secondary axis.** *Cell* 1995, **81**:85–94.

9. Katoh K, Standley DM: **MAFFT multiple sequence alignment software version 7: Improvements in performance and usability**. *Mol Biol Evol* 2013, **30**:772–780.

10. Stamatakis A: **RAxML version 8: A tool for phylogenetic analysis and post-analysis of large phylogenies**. *Bioinformatics* 2014, **30**:1312–1313.

11. Lartillot N, Lepage T, Blanquart S: **PhyloBayes 3: A Bayesian software package for phylogenetic reconstruction and molecular dating**. *Bioinformatics* 2009, **25**:2286–2288.
